# In silico design of an epitope-based vaccine ensemble for fasliolopsiasis

**DOI:** 10.3389/fgene.2024.1451853

**Published:** 2025-01-22

**Authors:** Ruchishree Konhar, Kanhu Charan Das, Aiboklang Nongrum, Rohan Raj Samal, Shailesh Kumar Sarangi, Devendra Kumar Biswal

**Affiliations:** ^1^ Informatics and Big Data, CSIR-Institute of Genomics and Integrative Biology, Delhi, India; ^2^ Academy of Scientific and Innovative Research, Ghaziabad, Uttar Pradesh, India; ^3^ Department of Zoology, North-Eastern Hill University, Shillong, Meghalaya, India; ^4^ Bioinformatics Centre, North-Eastern Hill University, Shillong, Meghalaya, India

**Keywords:** *Fasciolopsis buski*, TLR2, immune simulation, intestinal fluke, vaccine construct

## Abstract

**Introduction:**

Fasciolopsiasis, a food-borne intestinal disease is most common in Asia and the Indian subcontinent. Pigs are the reservoir host, and fasciolopsiasis is most widespread in locations where pigs are reared and aquatic plants are widely consumed. Human infection has been most commonly documented in China, Bangladesh, Southeast Asia, and parts of India. It predominates in school-age children, and significant worm burdens are not uncommon. The causal organism is *Fasciolopsis buski*, a giant intestinal fluke that infects humans and causes diarrhoea, fever, ascites, and intestinal blockage. The increasing prevalence of medication resistance and the necessity for an effective vaccination make controlling these diseases challenging.

**Methods:**

Over the last decade, we have achieved major advances in our understanding of intestinal fluke biology by in-depth interrogation and analysis of evolving *F. buski* omics datasets. The creation of large omics datasets for *F. buski* by our group has accelerated the discovery of key molecules involved in intestinal fluke biology, toxicity, and virulence that can be targeted for vaccine development. Finding successful vaccination antigen combinations from these huge number of genes/proteins in the available omics datasets is the key in combating these neglected tropical diseases. In the present study, we developed an *in silico* workflow to select antigens for composing a chimeric vaccine, which could be a significant technique for developing a fasciolopsiasis vaccine that prevents the parasite from causing serious harm.

**Results and discussion:**

This chimeric vaccine can now be tested experimentally and compared to other vaccine candidates to determine its potential influence on human health. Although the results are encouraging, additional validation is needed both *in vivo* and *in vitro*. Considering the extensive genetic data available for intestinal flukes that has expanded with technological advancements, we may need to reassess our methods and suggest a more sophisticated technique in the future for identifying vaccine molecules.

## 1 Introduction

*Fasciolopsis buski*, commonly known as the giant intestinal fluke, is one of the largest digenean trematode flatworms. This parasite causes fasciolopsiasis, which is prevalent in South Asian countries, including the Indian Subcontinent. It is important for veterinary and medical care because it affects both humans and pigs frequently. Dogs and rabbits can also contract the disease, but pigs serve as reservoirs. Severe infestation can be fatal if untreated, causing significant intestinal damage such as ulceration, erosion, abscesses, and haemorrhage (Biswal et al., 2013; Biswal et al., 2016a; 2018; Roberts et al., 2009). Notably, about 60% of infected individuals in India and mainland China remain asymptomatic.

*F. buski* thrives in regions where pig breeding is practiced and the consumption of aquatic plants like water chestnut, lotus, caltrop, and bamboo is prevalent. In India, this parasite is the sole documented species of its genus, prevalent in warm, moist areas such as the north-eastern part of India and exhibits morphological variation across geographic isolates (Roy and Tandon, 1993; Ridha et al., 2021; Hsu, 1964; Rajendran et al., 2019). The highest incidence rates are found in children, who typically ingest plants that have encysted metacercariae. The intestinal mucosa develops ulceration lesions and localised inflammation as a result of the parasite. There have been reports of an edematous state caused by protein-losing enteropathy in cases of severe infection (Sen-Hai and Mott, 1994).

The best way to control fasciolopsiasis is to avoid eating uncooked, water-based foods. Cooking aquatic vegetation or temporarily submerging plants or nuts in boiling water is typically enough to prevent infection. Also, restricting the use of human faeces as fertiliser and promoting modern pig rearing can help prevent fasciolopsiasis. This approach has been supported with large-scale screening protocols and improvements to water, sanitation and hygiene. Successful health education initiatives have reduced the transmission of this parasite in some endemic locations, such as Taiwan; but, in areas where fasciolopsiasis was assumed to be eliminated, such as Uttar Pradesh, in India, re-emerging infections has been observed (Bhatti et al., 2000).

Fasciolopsiasis is now managed in a way similar to other trematode diseases, with the goal of preventing transmission between mammalian hosts, including humans, and intermediate gastropod hosts, such as *Lymnaea* species. Praziquantel (PZQ) is a popular medicine due to its excellent efficacy in cases with fasciolopsiasis (Ridha et al., 2021; Taraschewski et al., 1986). Despite the efficiency of existing medications, the efficacy of vaccinations against helminth parasites like trematodes is mainly unknown. Recently, there has been growing concern regarding the development of anthelmintic resistance in fasciolopsiasis. Even though it has not become widespread, the appearance of resistance has led to a growing interest in other control/therapeutic methods, such as vaccines. At the time of writing, no vaccination has undergone human clinical trials or been authorised for commercial use in pigs.

Developing a vaccine for parasites like *F. buski* is therefore crucial due to rising drug resistance and the scarcity of new drugs (Nakagawa, 1922; Mas-coma et al., 2005). *F. buski*, like most helminths, has a complex eukaryotic multicellular life cycle, which complicates vaccination (Mas-coma et al., 2005; Sharma et al., 2015). The development of a vaccine would contribute to the WHO 2030 Roadmap for NTD goal of eliminating fasciolopsiasis as a public health problem (Malecela and Ducker, 2021).

While significant progress has been made in discovering possible vaccine molecules to reduce fasciolosis and fasciolopsiasis in livestock, it is reasonable to argue that the level of efficacy needed to bring the product to market has not yet been attained. Till date, three methods have been employed to find potential vaccine candidates against *F. hepatica* and *F. gigantica*: (i) examining antigens that exhibit cross-reactivity with sera from animals infected with other trematode infections; (ii) identifying orthologous antigens that have been identified as potential vaccine candidates in other species; and (iii) selecting antigens rationally that are crucial for liver fluke survival (Perera and Ndao, 2021). Many vaccine trials in livestock have assessed the efficacy of these potential antigens, including some single or chimeric experimental *F. hepatica* vaccines (Cox, 2002). Fatty acid binding proteins (FABP), glutathione S-transferase (GST), cathepsin L1 (CatL1), peroxiredoxin (Prx), and the gut-associated exopeptidase leucine aminopeptidase (LAP) have all been examined as potential leads. Similar studies on the intestinal fluke *F. buski* are lacking, and there is a need to design multi-subunit vaccines shared by *F. hepatica* and *F. gigantica*, which could lead to the development of a cost-effective vaccine with efficacy against both liver and intestinal flukes.

Understanding the functional role that each parasite molecule plays at each stage of the life cycle is crucial for developing new control methods. Over the last decade or so, the development of sequencing technologies and their application to the study of *Fasciola* species has paved the way for a global molecular understanding of the developmental biology of parasites. Comprehensive data on genomics, transcriptomics, proteomics, and glycomics has provided us with an in-depth knowledge of the parasite life cycle, from DNA synthesis and expression to the manufacture and post-translational modification of numerous essential parasite proteins. This understanding can be leveraged to build innovative control approaches. The availability of substantial data on the transcriptomes of intestinal flukes (*F. buski*) can help with this approach, as well as the identification of new vaccine targets (Biswal et al., 2018). It is important to note that mathematical modelling indicates that fasciolid vaccines, even if they do not provide complete protection, could significantly contribute to fluke control while also delaying the spread of anthelmintic resistance through reduced use of triclabendazole (Turner et al., 2016).

**FIGURE 1:**
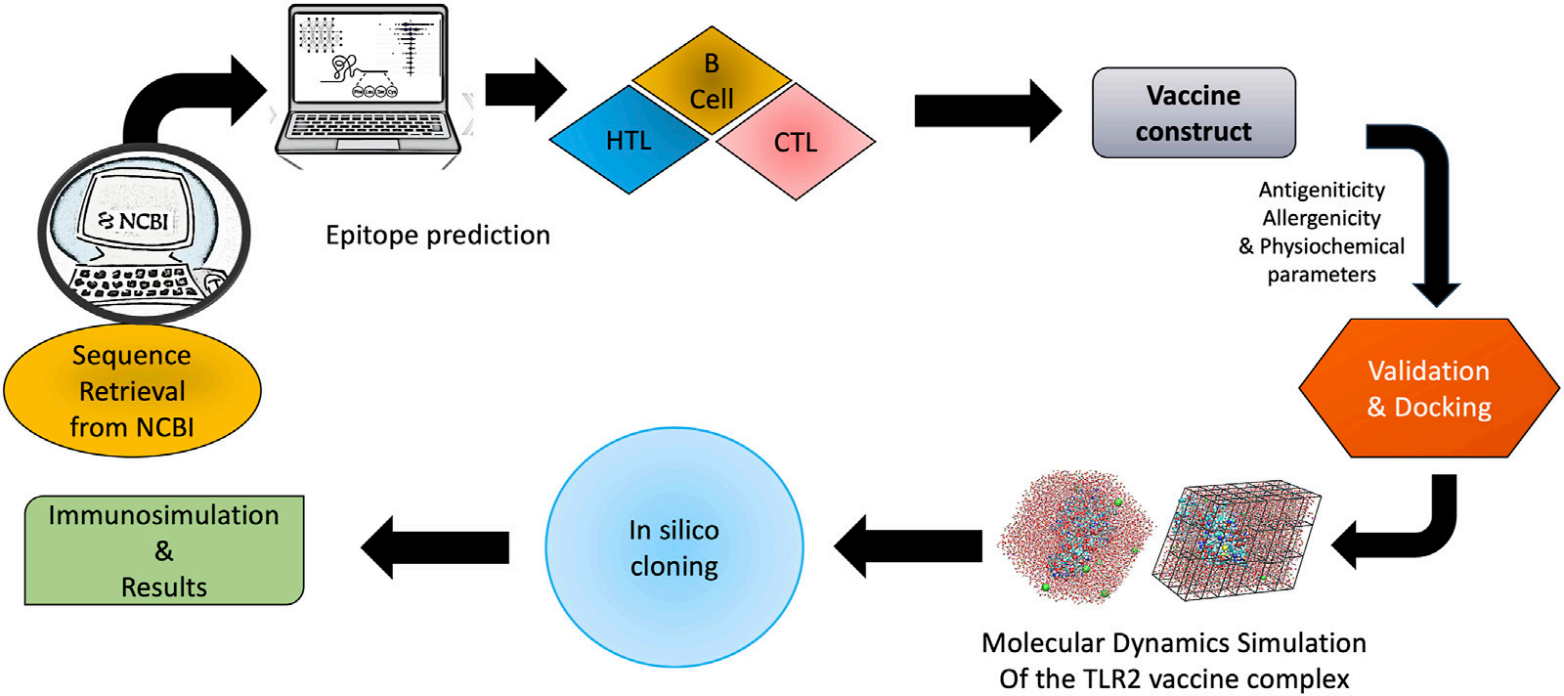
Schematic illustration of the workflow using B cell, CTL, and HTL epitopes followed by molecular docking, molecular dynamics simulation, and *in silico* cloning.

The aim of this study was to create and test a fasciolopsiasis vaccine for the first time utilising an *in silico* approach (Figure 1). This method has already been utilised to create vaccines against different pathogens for a variety of diseases, including SARS CoV-239 (Singh et al., 2020), Mycobacerium TB (Bibi et al., 2021), *Schistosoma mansoni* (Sanches et al., 2021), *F. hepatica*, *F. gigantica*, and *Onchocerca volvulus* (Shey et al., 2019). Previously, we report on the entire transcriptome and draft genome study of *F. buski*, which aided us in characterising some of its key biological properties and provided vast resources for the creation of a viable diagnostic system and anti-parasitic treatment strategies. In order to better combat these multicellular organisms, we have categorised these proteins into key biological processes (such as proteases, protease inhibitors, antioxidants, and immunomodulators) (Biswal et al., 2018) that can be combined to create a cocktail vaccine utilising the antigens found in *F. buski* to prevent infection in several hosts and obstruct the zoonotic transmission of the intestinal fluke. This could provide a way to get around the molecular redundancy and block or interfere with key parasitic processes.

## 2 Materials and methods

### 2.1 Selection and retrieval of vaccination targets

A fundamental understanding of the parasite biology has been achieved through the transcriptome data of the adult stage of this giant intestinal fluke, which was previously reported with Bioproject Accession: PRJNA212796 ID: 212,796, and is archived in the NCBI SRA: SRP028107, SRX327895, SRX326786, SRX316736, as well as in the North-East India Helminth Parasite Information Database (NEIHPID) developed by our group (Biswal et al., 2016b). This information is highly informative for the identification of potential drug targets and host-pathogen interaction studies.

The transcriptome of *F. buski* contains significant metabolic pathways, including glycan biosynthesis, lipid metabolism, carbohydrate metabolism, energy metabolism, amino acid metabolism, and nucleotide metabolism. The analysis of these pathways encompassed all genes related to glycolysis, the Krebs cycle, and fatty acid metabolism. The annotated genes were compared to those available in the Kyoto Encyclopedia of Genes and Genomes (KEGG) database using BLASTx. The mapped genes illustrated metabolic pathways involving key biomolecules, including carbohydrates, amino acids, and various other pathways. The glycolytic pathway includes all the essential enzymes, including hexokinase, enolase, pyruvate kinase, and lactate dehydrogenase.

**FIGURE 2:**
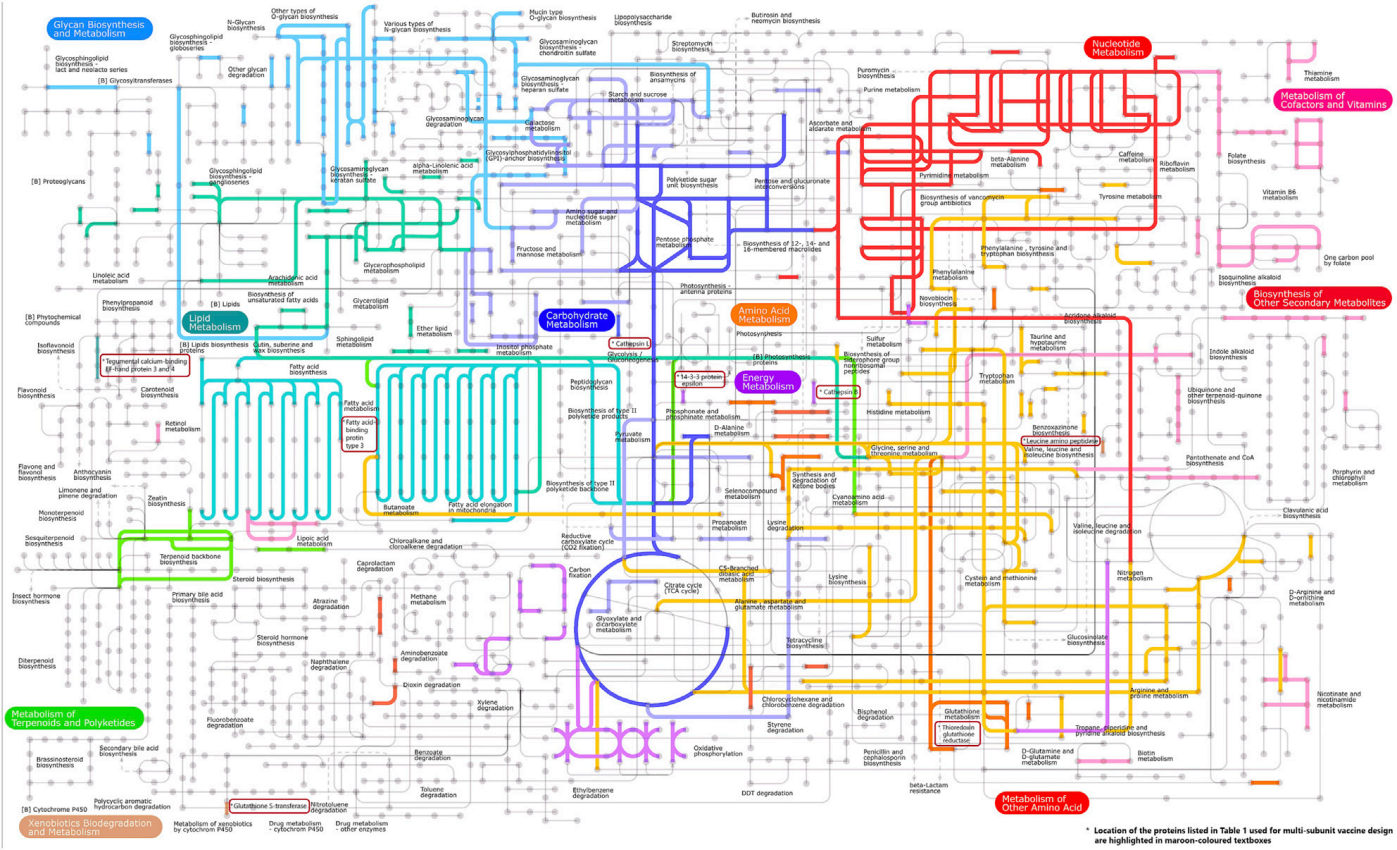
Important components of the metabolic pathways found in the transcriptome of F. buski. Colored edges indicate proteins that are homologous to any F. buski unigene. The positions of several key proteins mentioned in Table 1, which are utilized in the design of chimeric vaccines, are highlighted in maroon-colored textboxes. These proteins include Thioredoxin glutathione reductase (related to amino acid metabolism), Cathepsin B (associated with energy metabolism), Cathepsin L (involved in glycolysis/gluconeogenesis), Leucine amino peptidase (linked to amino acid metabolism), Fatty acid-binding protein type 3 (related to fatty acid metabolism), 14-3-3 protein epsilon (associated with energy metabolism), Glutathione S-transferase (involved in xenobiotics biodegradation and metabolism; Glutathione metabolism), and Tegumental calcium-binding EF-hand proteins 3 and 4 (related to lipid metabolism).

Pathway analysis is an approach for identifying vaccination targets by analysing the activity of confirmed gene sets that are biologically related. This strategy may be more effective since it focusses on gene sets rather than individual gene expression levels. The major components of metabolic pathways discovered in the *F. buski* transcriptome are depicted in Figure 2. Coloured edges indicate proteins that are homologous to any *F. buski* unigene. The skewed representation of enzymes in the pathway analysis have revealed insights into gene regulation and parasite biology, suggesting to a catabolic process and a possible reliance on their host for nourishment at several stages of their life cycle. However, pathway analysis has certain drawbacks, such as inadequate pathway information and the inability to include non-protein coding elements. Therefore, we relied on the combined approach of target proteins from previous studies (Williams et al., 2013; Smooker et al., 2010; Wijffels et al., 1994; Marcilla et al., 2008; Vicente et al., 2016; Chaithirayanon et al., 2006; Capron et al., 2001; Ehsan et al., 2021) and tallied that with pathway analysis from the transcriptome data manually. Some of the key proteins used in design of the chimeric vaccine viz. Thioredoxin glutathione reductase (Amino acid metabolism), Cathepsin B (Energy metabolism), Cathepsin L (Glycolysis/gluconeogenesis), Leucine amino peptidase (Amino acid metabolism), Fatty acid-binding protein type 3 (Fatty acid metabolism), 14-3-3 protein epsilon (Energy metabolism), Glutathione S-transferase (xenobiotics biodegradation and metabolism; Glutathione metabolism) and Tegumental calcium-binding EF-hand protein 3 & 4 (Lipid metabolism) are all indicated in Figure 2. The “names, “cellular locations,” and “functions” of each of the nine proteins used in the multi-subunit vaccine design and the rationale behind the choice of these vaccine targets are summarized in Table 1. A comprehensive description of these proteins is provided below.”

**TABLE 1:** List of proteins used for multi-subunit vaccine design showing their NCBI accessions and antigenicity Score.

Sl. No.	Protein name	Accession	Antigenicity score	Function	Cellular location
1	Thioredoxin glutathione reductase	KAA0194699.1	1.0428	Flavin Adenine Dinucleotide (FAD) Binding	Cytoplasmic
2	Cathepsin B	KAA0200776.1	1.0133	Intracellular Proteolysis	Extracellular, including cell wall
3	Cathepsin L	KAA0200004.1	1.0143	Peptide bond hydrolysis	Endoplasmic Reticulum
4	Leucine amino peptidase	KAA0200256.1	1.0378	Nuclear localization signal and Nuclear export signal	Cytoplasmic
5	Fatty acid-binding protein type 3	KAA0201253.1	0.9984	Transport of fatty acids and other lipophilic substances	Cytoplasmic
6	14-3-3 protein epsilon	KAA0194461.1	1.0191	Signal transduction and Cell proliferation	Cytoplasmic
7	Glutathione S-transferase	KAA0193008.1	1.0355	Glutathione transferase Catalysis	Cytoplasmic
8	Tegumental calcium-binding EF-hand protein 3	KAA0189127.1	1.0042	Calcium ion storage activity	Cytoplasmic
9	Tegumental calcium-binding EF-hand protein 4	KAA0189126.1	0.9997	Calcium ion storage activity	Cytoplasmic

Rationale behind the choice of proteins listed in Table 1 as vaccine targets.

A total of 9 different genes and respective proteins were selected (KAA0194699.1, KAA0200776.1, KAA0200004.1, KAA0200256.1, KAA0201253.1, KAA0194461.1, KAA0193008.1, KAA0189127.1, and KAA0189126.1) and obtained from NCBI in FASTA format. Additionally, the structure of TLR2 (PDB ID: 5D3I) was sourced from NCBI. The antigenicity of all proteins was predicted on the online server (http://imed.med.ucm.es/Tools/antigenic.pl).

#### 2.1.1 Thioredoxin glutathione reductase

A single enzyme, thioredoxin glutathione reductase (TGR), which is a fusion of a glutaredoxin (Grx) domain to classical thioredoxin reductase (TR) domains, provides electrons to oxidized glutathione (GSSG) and thioredoxin (Trx) in the simplified thiol-based redox system of Platyhelminth parasites. The use of TGR as a schistosomiasis medication target has been confirmed (Williams et al., 2013).

#### 2.1.2 Cathepsin B

Trematode (or fluke) cathepsin B proteases are widely distributed in juvenile and immature flukes. Cathepsin Bs are important for trematode biology, according to recent studies, mostly in Fasciola, that use RNA interference (RNAi), inhibitors, and vaccinations (Chantree et al., 2013). The damage caused by flukes when they infiltrate host tissues will be much reduced if these proteases are rendered inactive by chemotherapy or immunization, since they are primarily produced by infectious parasite stages. Cathepsin Bs are thus confirmed to be important strategic targets for fluke control (Smooker et al., 2010).

#### 2.1.3 Cathepsin L

Cathepsin L is a cysteine protease superfamily member that is extensively distributed in parasitic species. It plays critical roles in worm invasion, migration, food intake, moulting, and immunological evasion. Studies using pure native FheCL1 and FheCL2 as vaccines in sheep and cattle have demonstrated that these enzymes can produce strong anti-embryonation/hatch rate effects that would prevent the spread of parasites, as well as protection, ranging from 33% to 79%, against experimental challenges involving *F. hepatica* metacercariae (Wijffels et al., 1994).

#### 2.1.4 Leucine amino peptidase

The enzymes leucine aminopeptidase (LAP) and phosphoenolpyruvate carboxykinase have been identified by researchers as immunodominant antigens detected by sera from patients with fasciolosis using proteomics and immunoblotting techniques. Prior findings indicate that LAP elicits a robust and specific reaction, indicating its possible application in the serologic diagnosis of human *F. hepatica* infections (Marcilla et al., 2008).

#### 2.1.5 Fatty acid-binding protein type 3

Fatty-acid-binding proteins are among the substances that have been linked to interfering with the host response (FABPs). Approximately 15 kDa in size, FABPs are a multigenic family of small cytosolic proteins that primarily bind hydrophobic fatty acid ligands noncovalently. They exhibit distinct tissue distribution patterns and are linked to numerous important biological processes, such as the control of cellular lipid homeostasis, cell growth and differentiation, cellular signalling, gene transcription, and the immune response. In the gastrointestinal system of their hosts, helminth parasites thrive in an oxygen-poor environment where they are unable to synthesize most of their own lipids *de novo*, including cholesterol and long-chain fatty acids. Because helminths rely on the host’s fatty acids for intracellular lipid oxidation via FABP transportation, FABPs are essential to the biosynthesis of fatty acids and cholesterols in helminths. FABPs are an attractive choice as drug targets for novel anthelminthic vaccines because of their significant function in lipid oxidation, and their predominance in a variety of nematode, trematode, and cestode species (Vicente et al., 2016).

#### 2.1.6 14-3-3 protein epsilon

The 14-3-3 protein family comprises tiny, acidic proteins that operate as scaffolding, chaperones, and adaptors to regulate many intracellular signaling pathways. These proteins are involved in numerous physiological processes that are currently being studied for pharmaceutical intervention. Tested as prospective vaccine candidates in mice, the 14-3-3 proteins of *S. mansoni* and *Echinococcus multilocularis* show promise for use as defense mechanisms (Chaithirayanon et al., 2006). Clearly, to fully utilize the promise of 14-3-3-proteins as therapeutic targets, additional fundamental understanding as well as enhanced models and pharmacological candidates are needed.

#### 2.1.7 Glutathione S-transferase

Enzymes called glutathione S-transferases (GSTs) are found in parasites like trematodes and aid in the detoxification of foreign compounds in cells. GST is a target antigen that has been identified and molecularly cloned which has allowed for the demonstration of its vaccine potential in a number of animal species, including rodents, cattle, and primates, as well as the consistent demonstration of vaccination’s ability to decrease female worm fecundity and egg viability through the production of neutralizing antibodies (IgA and IgG). *S. haematobium* GST, Sh28GST, has been used in phase I and II clinical trials after encouraging preclinical research (Capron et al., 2001).

#### 2.1.8 Tegumental calcium-binding EF-hand protein 3 & 4

Tegumental proteins (TegPs) are members of the calcium-binding protein (CaBP) family, which also includes a dynein light chain-like domain at the C-terminus and two EF-hand motifs at the N-terminal domain. Numerous helminth parasites, such as *S. mansoni*, *E. multilocularis*, *Schistosoma hepatica*, *Schistosoma gigantica*, *Clonorchis sinensis*, and *Opisthorchis viverrini*, have been found to have CaBPs. CaBPs have been categorised into approximately 13 isoforms in *S. mansoni* and 4 isoforms in *F. hepatica* and *F. gigantica* (CaBP-1–4). Studies on the biochemical aspects of CaBPs from other parasites have revealed that, although the structural models of all family members are remarkably similar, these proteins’ dimerization, ion-binding, and drug-binding properties differ. Their crucial function in immune processes during host-parasite interactions may be demonstrated by this (Ehsan et al., 2021).

### 2.2 Epitope prediction

The B-cell epitopes were predicted using ABCPred tools (https://webs.iiitd.edu.in/raghava/abcpred/ABC_submission.html). The B-cell epitope database (BCIPEP) and an artificial neural network (ANN) were used for this (Saha and Raghava, 2006). The helper T-lymphocyte (HTL) epitopes were identified using MHC-II tools from the immune epitope database (IEDB, http://tools.iedb.org/mhcii/). Except for the alleles HLA-DPA1*01:03/DPB1*02:01 and HLA-DRB1*07:01, which encompass 99.9% of the global population [22,23], all parameters were left at their default values. Epitopes were chosen based on their lowest percentile rank and IC50. Cytotoxic T-lymphocyte (CTL) epitopes were predicted using the NetCTL 1.2 tools (http://www.cbs.dtu.dk/services/NetCTL/), a default threshold value of 0.75 and three allele supertypes (A2, A3, and B7) with a population coverage of 88.3% were configured in the tools. IFN-γ inducing epitope prediction was used to identify positive and negative inducers (http://crdd.osdd.net/raghava/ifnepitope/predict.php) (Dhanda et al., 2013). The parameters were maintained at their default settings, with the exception of the Motif and SVM hybrid, as well as IFN-γ versus other cytokine model.

### 2.3 Toxicity and epitope identity assessment

The ToxinPred module (http://crdd.osdd.net/raghava/toxinpred/multi.submit.php) was used to determine the toxicity of the chosen epitopes and show whether they were toxic or not. Standard parameters like Sensitivity, Specificity, Accuracy, and Matthew’s correlation coefficient (MCC) were taken into consideration while assessing the performance of the models. Default physiochemical properties (Hydrophobicity, Hydrophilicity Charge and Molecular Weight) were considered for predicting the toxicity of the epitopes used in this study. All parameters have been set to their default values (Gupta et al., 2013). Furthermore, epitopes were tested for identity in humans and pigs. These epitopes were run through a BLASTp (Basic Local Alignment Search Tool protein) search (https://blast.ncbi.nlm.nih.gov/Blast.cgi) (Altschul et al., 1990) and were cross-checked using the Multiple Peptide Match tool in PIR (https://research.bioinformatics.udel.edu/peptidematch/batchpeptidematch.jsp) (Wu et al., 2003). Epitopes were matched against the human and pig proteomes using the UniProt/SwissProt option, which included the various isoforms. Epitopes anticipated to be harmful or identified in humans or pigs were excluded, and a new epitope was chosen for testing.

### 2.4 Vaccine design

The vaccine was designed by using the adjuvant followed by B-cell, CTL and HTL epitopes joined by KK, AAY and GPGPG linkers respectively. Linkers were employed to arrange the amino-acid into favourable conformations. An Adjuvant is employed to enhance the immunogenicity of the corresponding vaccine.

### 2.5 Antigenicity, allergenicity and physiochemical parameters

The capacity to bind B-cell or T-cell receptors is referred to as antigenicity. Tools ANTIGENpro and VaxiJen (Doytchinova and Flower, 2007a; Doytchinova and Flower, 2007b) were used to assess the vaccine’s antigenicity. The Allertop (https://www.ddg-pharmfac.net/AllerTOP/) (Dimitrov et al., 2014) and Algpred (https://webs.iiitd.edu.in/raghava/algpred/submission.html) (Saha and Raghava, 2006) servers were used to evaluate the allergenicity of the vaccine design. Models of bacteria and parasites were employed with the VaxiJen v2.0 server, and a 0.7 threshold was applied in each case. Using the ToxinPred site and the previously described approach, the toxicity of vaccines was assessed. It is anticipated that the multi-epitope vaccination will be non-toxic, non-allergic, and antigenic. With the Expasy ProtParam tool (https://web.expasy.org/protparam/) (Wilkins et al., 1999), the construct’s physicochemical parameters were examined. Molecular weight, *in vitro* and *in vivo* half-lives, instability index, theoretical isoelectric point (pI), aliphatic index, and grand average of hydropathicity (GRAVY) were among the attributes that were analysed. The vaccine solubility upon overexpression in *E. coli* was assessed with SOLPro (http://scrat.ch.proteomics.ics.uci.edu/) (Magnan et al., 2009). The tools PSIPRED 4.0 (http://bioinf.cs.ucl.ac.uk/psipred/) (Buchan and Jones, 2019) and RaptorX Property Prediction (https://github.com/j3xugit/RaptorX-3DModeling) (Wang et al., 2016) were used to predict the vaccine secondary structure (2D).

### 2.6 Prediction of structure of the vaccine, refinement, validation and docking

The I-Tasser server (http://raptorx.uchicago.edu/StructurePrediction/predict/) was used to predict the construct vaccine’s tertiary structure (Källberg et al., 2012). ProsA was used for structural validation, and Pdbsum was used to create the Ramachandran plot (Wiederstein and Sippl, 2007; Laskowski et al., 2018). The model was further optimised using the Galaxy server (Heo et al., 2013), and RMSD was used to determine which model was the best. TLR2 (PDBID: 5D3I) was obtained from the PDB. ClusPro (http://nrc.bu.edu/cluster) was used for docking, and among the generated models, the most optimal model was chosen based on binding energy for the vaccine construct (Kozakov et al., 2013; Kozakov et al., 2017). Further the Staphylococcal superantigen-like protein 3 (SSL3) complex with TLR was excluded because it interacts with the TLR2 complex through lipopeptides and blocks the binding site of the TLR2 ligand, interfering with the formation of heterodimers between TLR2 and TLR1. Numerous publications have detailed the ligand-induced interactions of TLR2 with other pattern recognition receptors, including integrins, scavenger receptors, CD14, and a variety of other receptors, all of which are crucial for TLR2 activity. These “information-driven” approaches are successful in TLR2-complex docking carried out by ClusPro because of the conserved aspects of the binding interface. Of all the models generated from ClusPro, the most effective model in terms of binding energy to bind the vaccine construct was chosen. The binding residue information was supplied as attractive residues. These information in the TLR2, following residues that are involved in SSL3 binding viz. ASP294, TYR326, LEU328, SER329, TYR332, SER354, ASP294 and ASP327, Phe349, Leu350, Gln375, Tyr376, Asn379, Phe349, Leu350, Gln375, Tyr376, and Asn379, His358, Leu324, Tyr326 were taken based on previously published experimental data. To specify attraction, we entered the residues that are believed to participate in the binding into one or both sides of the interface. In the docking calculations, an extra attractive force was applied to the selected residues. The following whole adjuvant sequence (LprA) was inserted in vaccine construct as shown below (LprA being agonist of TLR2).

(MKHPPCSVVAAATAILAVVLAIGGCSTEGDAGKASDTAATASNGDAAMLLKQATDAMRKVTGMHVRLAVTGDVPNLRVTKLEGDISNTPQTVATGSATLLVGNKSEDAKFVYVDGHLYSDLGQPGTYTDFGNGASIYNVSVLLDPNKGLANLLANLKDASVAGSQQADGVATTKITGNSSADDIATLAGSRLTSEDVKTVPTTVWIASDGSSHLVQIQIAPTKDTSVTLTMSDWGKQVTATKPV).

### 2.7 Molecular dynamics simulation of the TLR2-vaccine complex

The molecular dynamics simulation (MDS) for the vaccine was performed on Ubuntu 2020.1 gromacs version. The docked complex was immersed in a TIP3P water model and the AMBER99SB force field. The Genion tool was employed to add 7 Cl^−^ to achieve system neutralization and to mitigate steric clashes of the system they were kept in energy minimization and force below 1,000 kj mol^−1^ nm^−1^. Long-range electrostatics were calculated using the Particle Mesh Ewald (PME) method, while Lennard-Jones and coulomb were computed with a cut-off distance of 1.0 nm. Bond lengths were constrained using the LINCS algorithm, and the SHAKE algorithm was employed to find out water bond. Following energy minimization, the system was equilibrated at 1 ns of NVT (constant number of particles, volume, and temperature) and NPT (constant number of particles, pressure, and temperature), and the system was run for 100 ns. Analysis tools g_rms, g_rmsf, and g_hbonds were used to calculate the root mean square deviation (RMSD), root mean square fluctuation (RMSF), and number of hydrogen bonds, respectively (Abraham et al., 2015).

### 2.8 In-silico cloning

Codon optimisation is critical for performing *in silico* cloning in a bacterial expression system. This procedure was carried out using the Java Codon Adaptation Tool (http://www.jcat.de/), with the *E. coli* K12 strain as the host organism. The parameters selected were “avoid Rho independent terminators,” “avoid prokaryotic ribosome binding sites,” and “avoid restriction enzyme cleavage sites.” The optimal Codon Adaptation Index (CAI) and GC concentration are 0.8%–1.0% and 30%–70%, respectively. The optimised vaccination sequence was then reversed to include XhoI and BamHI restriction sites at the N and C terminals. The integration of the sequence was assisted using the pET28a(+) vector (d'Aubenton Carafa et al., 1990; Carbone et al., 2003; Grote et al., 2005).

### 2.9 Immune simulation for vaccine efficacy

In silico simulations of the human immune response to the chimeric molecule were conducted using the C-IMMSIM web server (https://wwwold.iac.rm.cnr.it/∼filippo/c-immsim/index.html). We administered three doses, 4 weeks apart. To mimic 1,050 simulation steps, injections of 1,000 vaccine proteins were administered 4 weeks apart at 1, 84, and 168 time-steps (each time-step is equivalent to 8 h in real life). The remaining parameters were kept at their defaults (Greenwood, 2014).

## 3 Results

### 3.1 Selected vaccination targets and respective epitopes for vaccine design

We have focussed on chimeric molecule vaccine development against fasciolopsis by considering those molecules which are present at the host–parasite interface, specifically proteins within the parasite’s tegument and gut, extracellular vesicles, which appear in the parasite’s secretome and play a critical role in host invasion, host immune modulation/manipulation, and parasite survival. Transcriptomic and proteomic studies have increased our understanding of these important proteins by unmasking their expression and secretory profiles in the various *F. buski* developmental life cycle stages. The antigenicity all the nine sequences for *F. buski* as shown in Table 1, was assessed based on the antigenic score (Table 1) to boost the immunogenicity of the protein TLR-2 agonist Lipoprotein LprA (P9WK55) (Figure 1).

### 3.2 Anticipated immune properties of the chimeric molecule vaccination

The ABCpred server was used to predict B-cell epitopes, and the top nine epitopes were chosen from nine proteins (Table 2). All protein sequences were then submitted to IEDB MHC-II for HTL epitope prediction. Nine epitopes were selected from the proteins based on their low percentile score and IC50 value. Notably, epitopes with high binding affinity had the lowest percentile values (Table 3). Nine epitopes were selected from the proteins based on their low percentile score and IC50 value. Notably, epitopes with high binding affinity had the lowest percentile values (Table 3). The parameters remained unchanged, with the exception of a selection of alleles, LA-DPA1*01:03/DPB1*02:01 and HLA-DRB1*07:01, based on the disease’s geographical distribution. The NetCTL1.2 server was utilised to identify the CTL epitopes. The vaccine design used the A2, A3, and B7 supertypes of the MHC class, out of 27. The default threshold of 0.75 was used, and the epitopes with the highest scores were picked, totalling twenty-seven (Table 4). The vaccine was ultimately built around three B-cell, three HTL, and nine CTL epitopes. All epitopes tested positive for IFN-γ production (Table 3). Furthermore, the toxicity of the epitopes was determined using the ToxinPred programme, which demonstrated that all epitopes were non-toxic (Table 5).

**TABLE 2:** List of selected B-cell epitopes based on highest scores for the proteins sequences used in this study.

Sl. No.	Accession	Epitope	Score
1	KAA0194699.1	REGMEDSTHFGWKFDP	0.93
2	KAA0200776.1	EGYNITYPEDRHYAKI	0.93
3	KAA0200004.1	TGKEDTLKRMIAEQGP	0.94
4	KAA0200256.1	STVRKEDYEFNRGKTE	0.93
5	KAA0201253.1	RTEINTYPEFASCILP	0.92
6	KAA0194461.1	VLMQGDYYRYCAEVAD	0.92
7	KAA0193008.1	YGKEDGAKWFGEKFNL	0.95
8	KAA0189127.1	YWMNFTHEPFMSIQFK	0.95
9	KAA0189126.1	ESLIDWFMELDKNNDE	0.92

**TABLE 3:** List of selected HTL epitopes for the proteins sequences under study and their IFN-γ inducing properties.

Sl. No.	Protein name	Accession	Epitope	Score	IFN-γ
1	Thioredoxin glutathione reductase	KAA0194699.1	YCHAVSYLFSWFLAG	0.15	Positive
2	Cathepsin B	KAA0200776.1	KLRSNELFLSRLGAH	4.10	Positive
3	Cathepsin L	KAA0200004.1	LNQFTDMTFEEFKAK	0.91	Positive
4	Leucine amino peptidase	KAA0200256.1	KNPFLFTVATLTGHV	0.47	Positive
5	Fatty acid-binding protein type 3	KAA0201253.1	KMTMRTVSALKTTTI	4.00	Positive
6	14-3-3 protein epsilon	KAA0194461.1	LGLALNFSVFYYEIK	0.49	Positive
7	Glutathione S-transferase	KAA0193008.1	DEKVKLTQSVAILRY	0.40	Positive
8	Tegumental calcium-binding EF-hand protein 3	KAA0189127.1	MNFTHEPFMSIQFKY	0.67	Positive
9	Tegumental calcium-binding EF-hand protein 4	KAA0189126.1	MQFSHEPFLSIQFRF	0.10	Positive

**TABLE 4:** List of selected CTL epitopes for proteins sequences used in this study.

Sl. No.		Epitope	Score
1	A2	YINAFAELV	1.1324
2		FLSRLGAHM	0.9422
3		FLSRLGAHM	0.91
4		AMLGAYHAL	1.0798
5		KLIESTKPV	1.0121
6		QLLRDNLTL	1.0435
7		FMVYELLDV	1.3469
8		QLISLFLEI	1.2611
9		SLIDWFMEL	1.5533
10	A3	LMHQAALLR	1.1324
11		GIYFHSWGK	1.4230
12		GIYFHSWGK	1.4230
13		MTLASGLDK	1.2649
14		TMRTVSALK	1.3955
15		ALNFSVFYY	1.6024
16		KMWSHFLDR	1.5235
17		RTNQQTELK	1.2565
18		GSYWMQFSH	0.7655
19	B7	LPYPPGKTL	1.39
20		VPANEQQIM	0.9346
21		VPANEQQIM	0.9346
22		APLLYLGAL	1.4628
23		MTMRTVSAL	1.1460
24		HPIRLGLAL	1.5910
25		SPDFEKLKV	0.8435
26		MPVERQEEV	1.3211
27		IPAEKIDEW	0.7018

**TABLE 5:** Toxicity of the epitopes was assessed using the ToxinPred tool.

Sl. No.	Epitope	Toxicity
1	REGMEDSTHFGWKFDP	Non-Toxic
2	EGYNITYPEDRHYAKI	Non-Toxic
3	TGKEDTLKRMIAEQGP	Non-Toxic
4	STVRKEDYEFNRGKTE	Non-Toxic
5	RTEINTYPEFASCILP	Non-Toxic
6	VLMQGDYYRYCAEVAD	Non-Toxic
7	YGKEDGAKWFGEKFNL	Non-Toxic
8	YWMNFTHEPFMSIQFK	Non-Toxic
9	ESLIDWFMELDKNNDE	Non-Toxic
10	YCHAVSYLFSWFLAG	Non-Toxic
11	KLRSNELFLSRLGAH	Non-Toxic
12	LNQFTDMTFEEFKAK	Non-Toxic
13	KNPFLFTVATLTGHV	Non-Toxic
14	KMTMRTVSALKTTTI	Non-Toxic
15	LGLALNFSVFYYEIK	Non-Toxic
16	DEKVKLTQSVAILRY	Non-Toxic
17	MNFTHEPFMSIQFKY	Non-Toxic
18	MQFSHEPFLSIQFRF	Non-Toxic
19	YINAFAELV	Non-Toxic
20	FLSRLGAHM	Non-Toxic
21	FLSRLGAHM	Non-Toxic
22	AMLGAYHAL	Non-Toxic
23	KLIESTKPV	Non-Toxic
24	QLLRDNLTL	Non-Toxic
25	FMVYELLDV	Non-Toxic
26	QLISLFLEI	Non-Toxic
27	SLIDWFMEL	Non-Toxic
28	LMHQAALLR	Non-Toxic
29	GIYFHSWGK	Non-Toxic
30	GIYFHSWGK	Non-Toxic
31	MTLASGLDK	Non-Toxic
32	TMRTVSALK	Non-Toxic
33	ALNFSVFYY	Non-Toxic
34	KMWSHFLDR	Non-Toxic
35	RTNQQTELK	Non-Toxic
36	GSYWMQFSH	Non-Toxic
37	LPYPPGKTL	Non-Toxic
38	VPANEQQIM	Non-Toxic
39	VPANEQQIM	Non-Toxic
40	APLLYLGAL	Non-Toxic
41	MTMRTVSAL	Non-Toxic
42	HPIRLGLAL	Non-Toxic
43	SPDFEKLKV	Non-Toxic
44	MPVERQEEV	Non-Toxic
45	IPAEKIDEW	Non-Toxic

### 3.3 Methodology for vaccine construct

To construct the vaccine, we designed the most favourable CTL, HTL, and B-Cell epitopes using AAY, GPGPG, and KK linkers, respectively. Furthermore, an EAAAK linker was used to bind Lipoprotein LprA Adjuvant (NCBI ID: P9WK55) to the vaccine’s the N-terminal. The vaccine’s structure comprises of nine HTL epitopes, twenty-seven CTL epitopes, and nine B-Cell epitopes derived from the target protein sequences. The use of a TLR-2 agonist, Lipoprotein LprA (P9WK55), increased the vaccine’s immunogenicity due to its adjuvant qualities. All of the identified epitopes from B-cell (9-mers), HTL (9-mers), and CTL (27-mers) were linked using particular linkers EAAAK between adjuvant and epitopes. After evaluating and comparing several structures, we determined the vaccine’s final structure with 901 amino acids. The final recombinant vaccine was analysed for subsequent evaluations.

### 3.4 Antigenicity, allergenicity and physiochemical properties

The predicted vaccine design had antigenicity values of 0.6244 and 0.5541 in the ANTIGENpro and Vaxijen servers, respectively. Allertop and Algpred determined the allergenicity of the construct vaccine, which indicated that it is non-allergenic. In the allergenicity testing utilising Allergen FP tools, the protein with the highest Tanimoto similarity index is uniprotkb Q2RB59 (0.85). ProtParam, an online programme, was used to predict the vaccine construct’s physiochemical properties. The molecular weight of the 901 amino acid protein was 98.71 kDa, and its theoretical PI was 8.74. The vaccine construct had an instability score of 27.36 and an aliphatic index of 76.93, which confirmed the protein’s thermostability. The GRAVY score of −0.136 indicates the protein’s hydrophilic nature. Therefore, the resulting vaccine is extremely acidic, thermostable, and hydrophilic, making it suitable to undergo subsequent efficacy testing.

### 3.5 Vaccine modelling and validation

**FIGURE 3:**
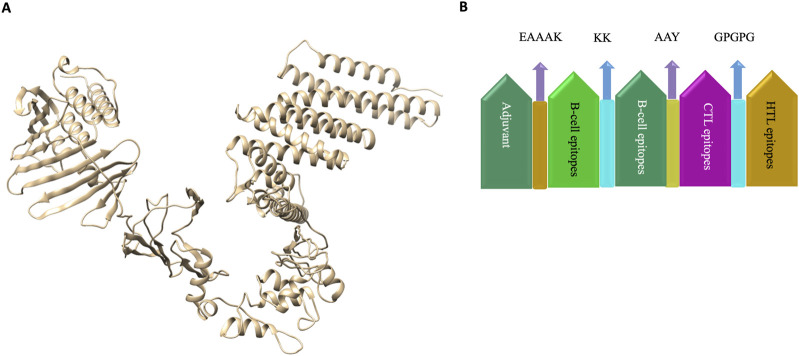
**(A)** 3D structure of vaccine construct. **(B)**. Vaccine was designed by using the adjuvant followed by B-cell, CTL and HTL epitopes joined by KK, AAY and GPGPG linkers respectively. Linkers were employed to arrange the amino-acid into favourable conformations. An Adjuvant is employed to enhance the immunogenicity of the corresponding vaccine.

**FIGURE 4:**
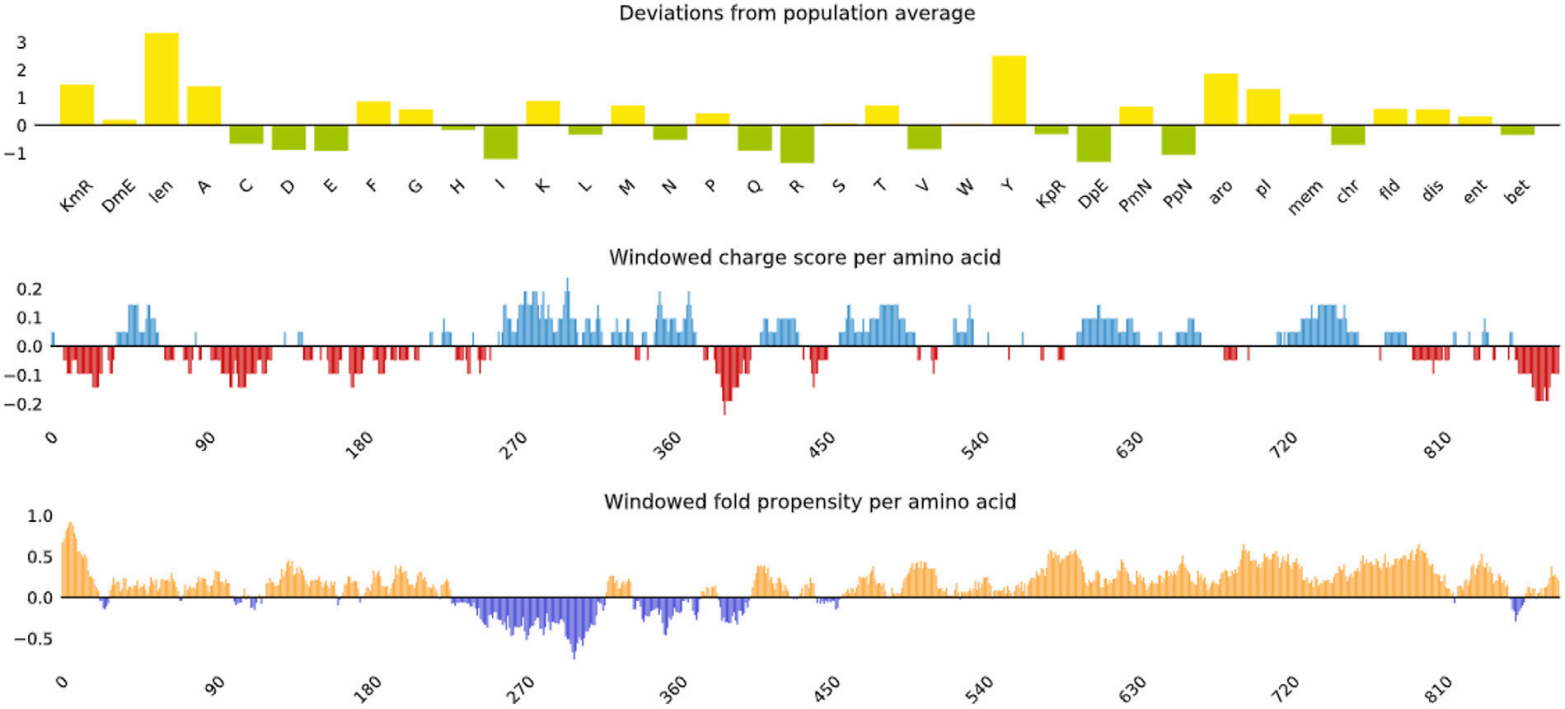
Vaccine construct solubility as determined by the protein sol server that showed deviation population average, charge per amino acid and fold propensity.

The 3-D model of the vaccine construct was predicted using Raptor-X (Figure 3) and was subjected to additional refinement on the Galaxy server to acquire a valid structure. The Ramachandran plot derived from the model was 89.2%, 8.2%, 1.8%, and 0.8% in the most favoured regions (Supplementary Figure 1A), additional allowed region, generously allowed region, disallowed region. Following refinement, the validate structure exhibit 91.5% in most favoured regions, 7.6% in additional allowed region, 0.1% in generously allowed region, 0.5% in disallowed region (Supplementary Figure 1B). The ProsA server display (Z-score was −10.4) (Supplementary Figures 2A, B), indicating the qualitative characteristics of the vaccine construct. The energy plot indicated that the initial few sequences exhibit slightly elevated energy levels, followed by a substantial decrease, suggesting that the protein is thermodynamically stable (Supplementary Figure 2C). Protein Sol server was performed that showed deviation population average, charge per amino acid and fold propensity (Figure 4). Secondary structure was predicted by pdbsum server (Supplementary Figures 3A, B).

### 3.6 Molecular docking with vaccine and TLR2

**FIGURE 5:**
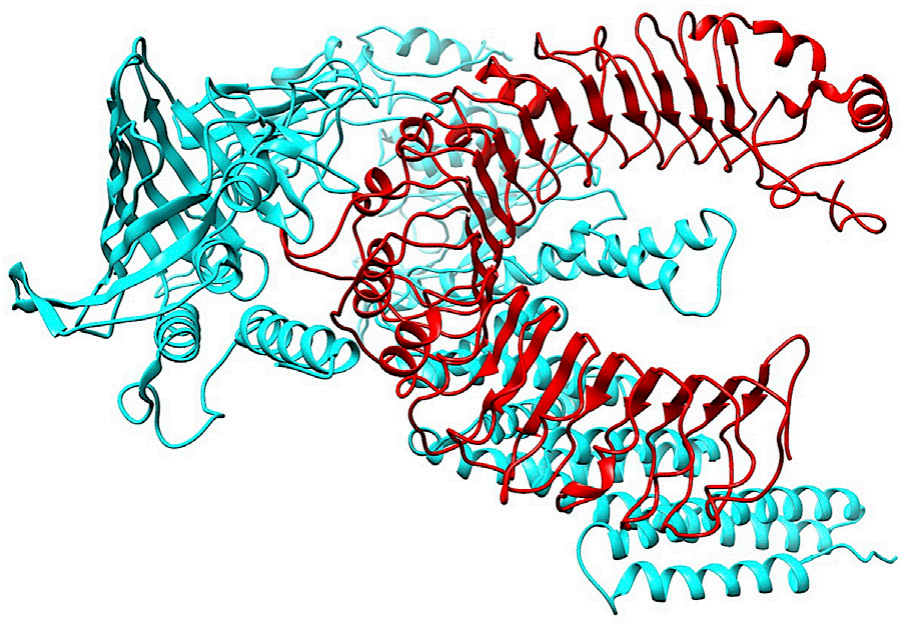
Molecular Docking of TLR2 and vaccine complex. To investigate the relationship between the TLR2 (5D3I) and the vaccine construct, molecular docking was used. Of the sixteen models that were generated, the optimal model for the dynamics investigation between TLR2 and vaccination and interaction was selected based on the lowest binding energy of −1,287.2 kJ.mol^−1^.

**FIGURE 6:**
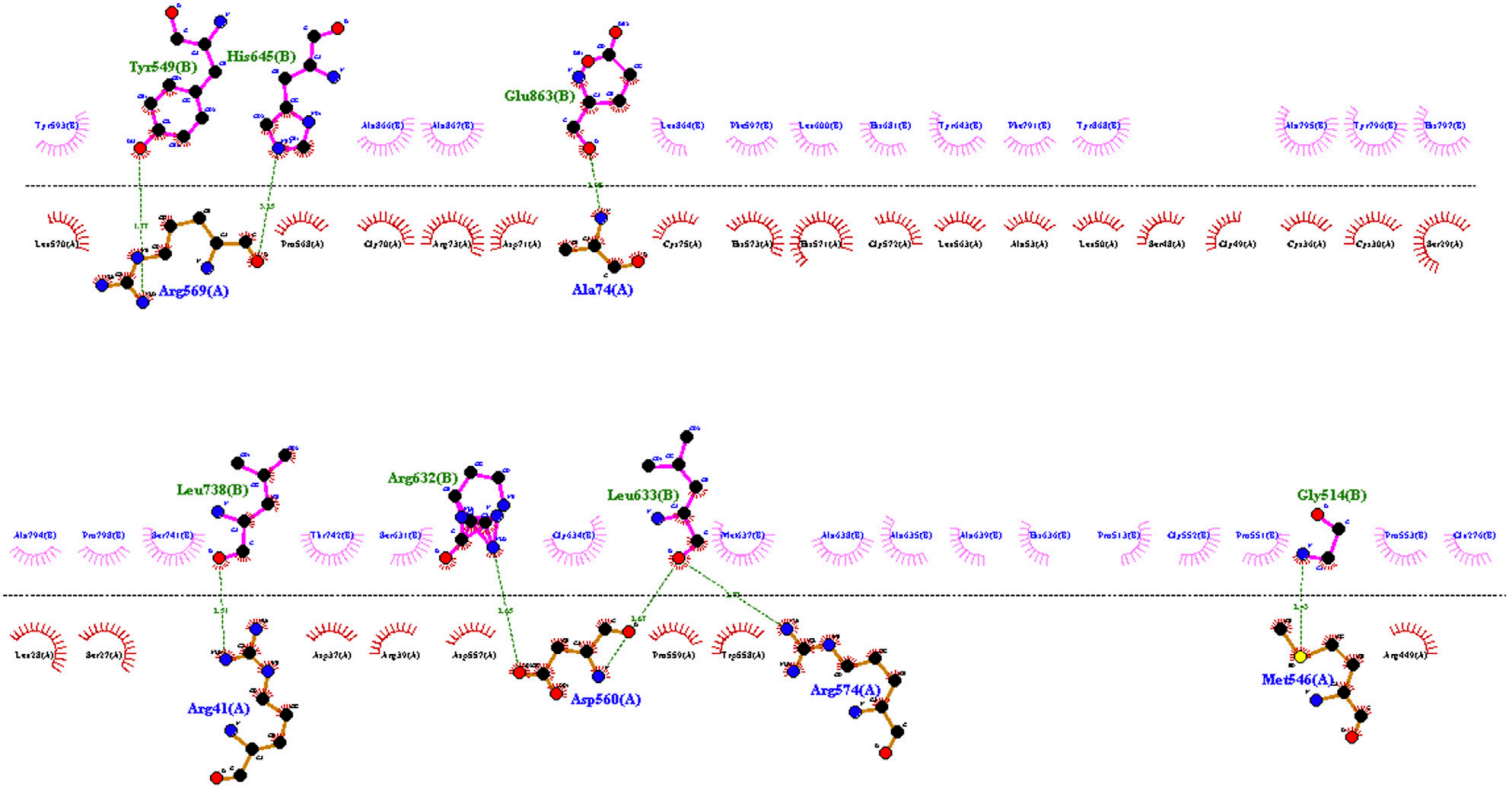
Interaction between TLR2 and the vaccine construct. The interaction map of the vaccine construct with chains A and B from TLR2. Hydrogen bonds are shown by dashed (green) lines with the length of the bond printed in the middle.

**FIGURE 7:**
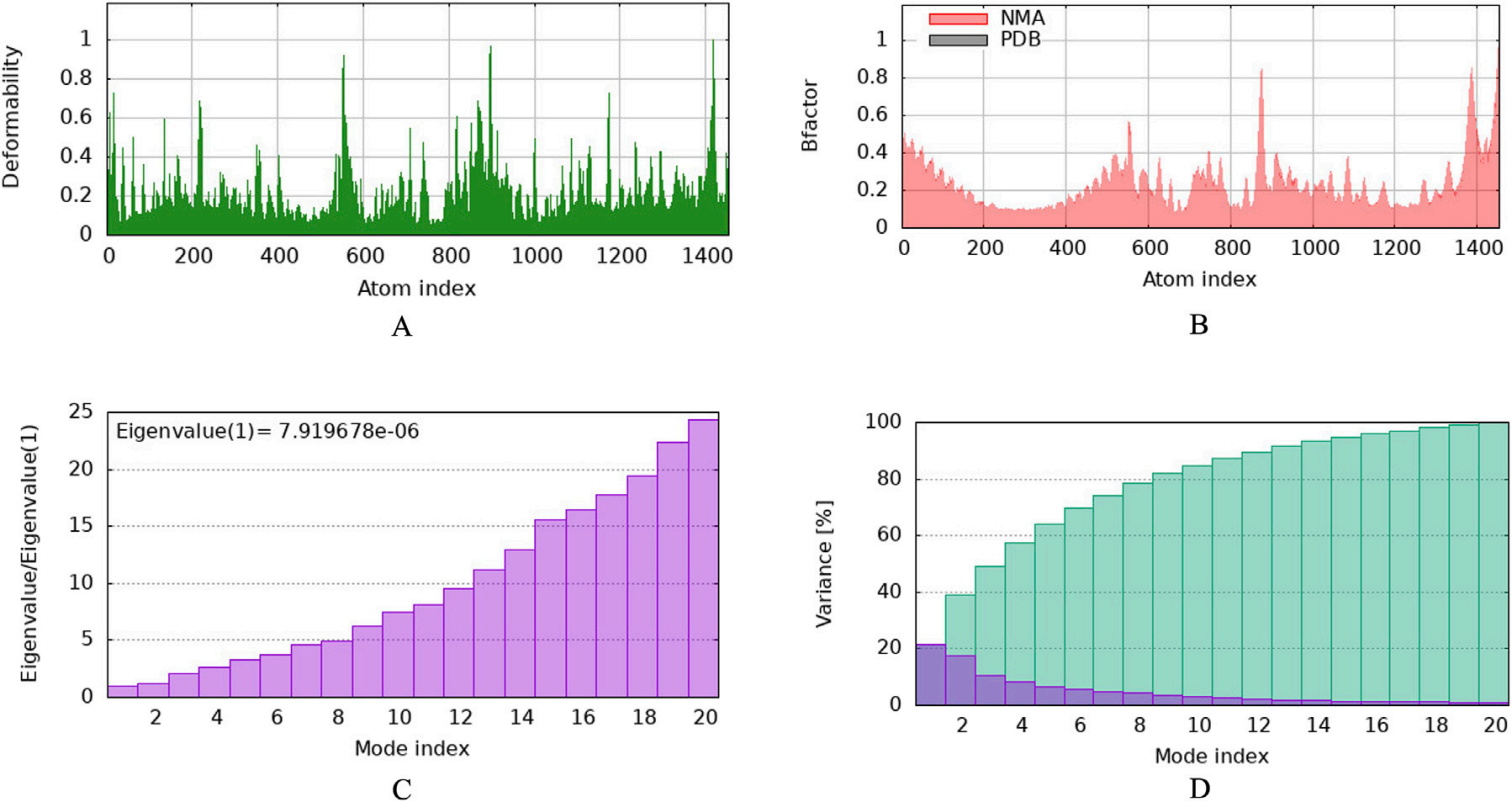
Molecular dynamics simulation of vaccine protein complex. Stability of the vaccine depicted via graphical representation. **(A)** Deformity vs. Atomic index of the vaccine construct, **(B)** Bfactor vs. Atomic index of the vaccine, **(C)** Eigenvalue vs. Atomic index of the vaccine construct, **(D)** Variance vs. Atomic index of the vaccine construct.

**FIGURE 8:**
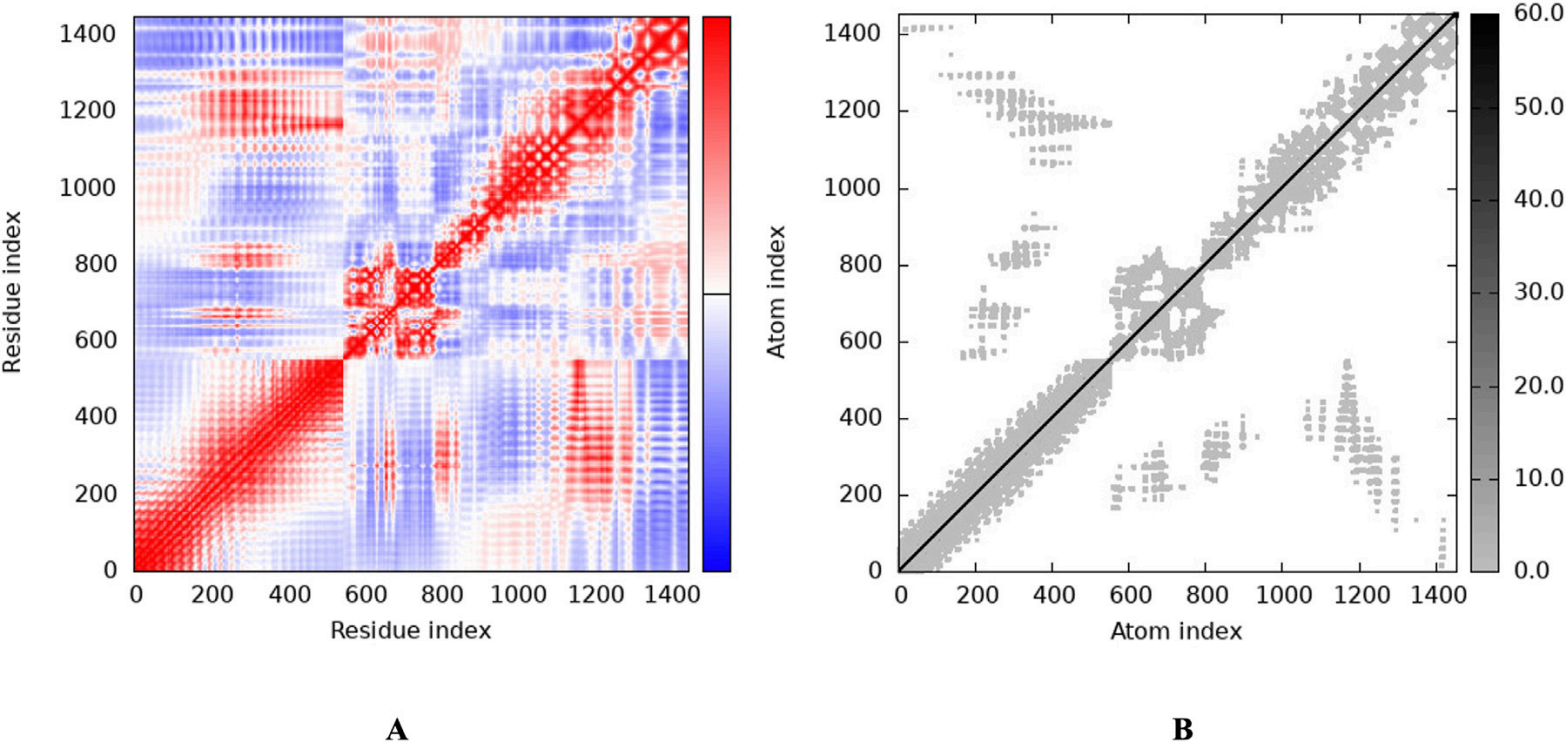
Stability of the vaccine via graphical representation. **(A)** Representation of the Residue index (covariance), **(B)** Representation of the Atomic index (elastic network analysis).

To investigate the relationship between the TLR2 (5D3I) and the vaccine construct, molecular docking was used. Out of the sixteen models that were generated, the optimal model for the dynamics investigation between TLR2 and vaccination (Figure 5) and interaction (Figure 6) was selected based on the lowest binding energy of −1,287.2 kJ.mol^−1^. For the complicated profiles of mobility (NMA B-factors), deformability, eigenvalues, covariance map, and linkage matrix, iMODS server was used (Figures 7A–D, 8A).

### 3.7 Dynamics study of the complex

**FIGURE 9:**
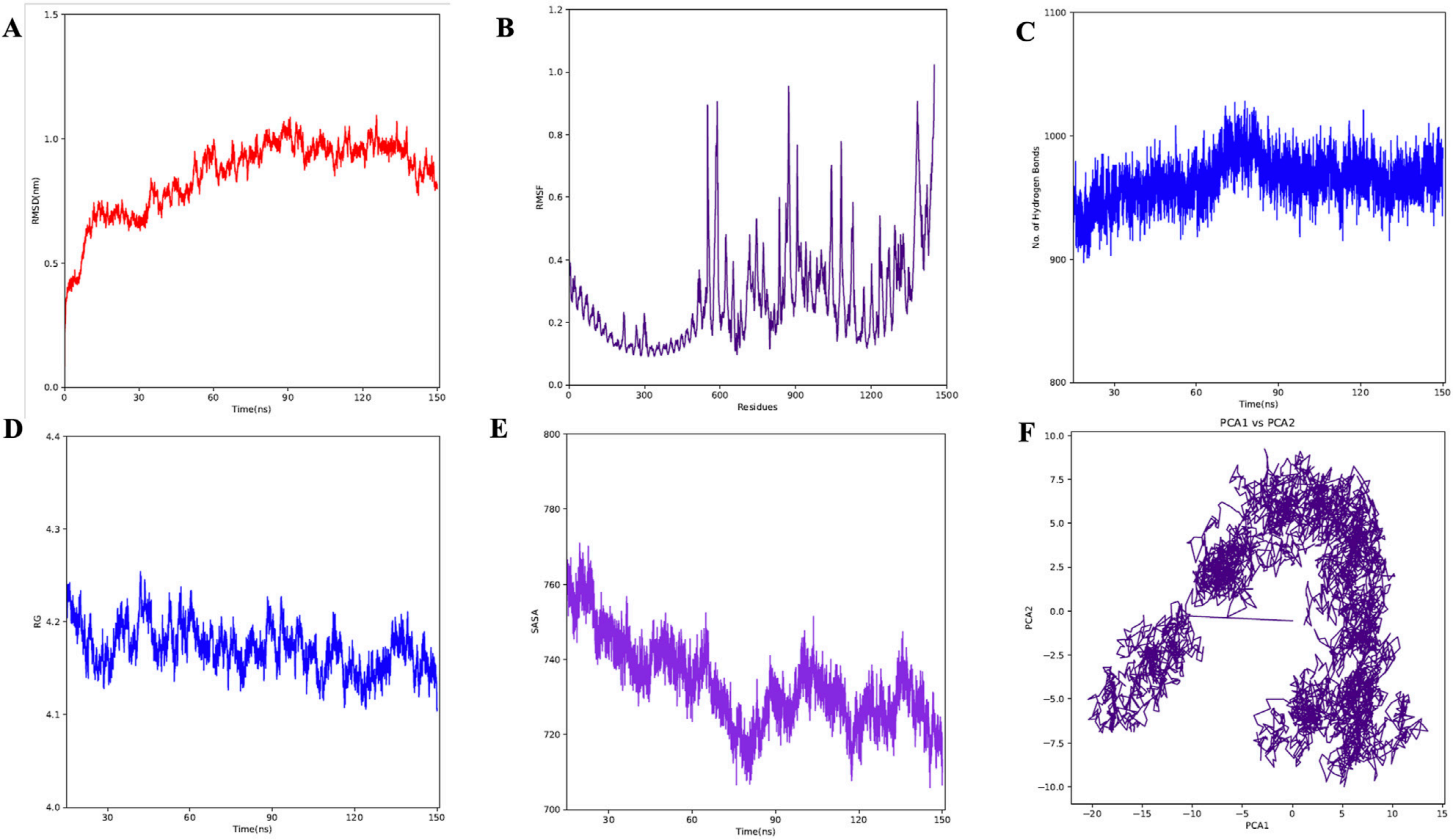
MD simulation of complex. **(A)** RMSD for the amino acid backbone of the complex, **(B)** RMSF of amino acids side chain of the complex **(C)** Number of hydrogen bonds formed during last 50 ns, **(D)** Radius gyration (R_g_) of the complex, **(E)** Surface area (SASA) of the vaccine construct, **(F)** Principal Component Analysis (PCA) of the vaccine construct.

A molecular dynamics simulation (MDS) of the TLR2-vaccine complex was run for 150 ns to evaluate its thermodynamic properties. The parameters RMSD, RMSF, Rg, and hydrogen bonds were calculated. After 10 ns, the system reached stability. Analysis of Cα atoms revealed an average RMSD of 0.84 nm ± 0.15 (Figure 9A). The RMSF revealed residue variations, with an average value of 0.27 ± 0.15 nm (Figure 9B). The protein-protein complex’s stability is indicated by its hydrogen bonds, which averaged 963.67 ± 19.15 (Figure 9C). Rg values, which indicate protein compactness, averaged 4.17 ± 0.02 nm (Figure 9D).

### 3.8 In-silico cloning into pET28 (+) vector

**FIGURE 10:**
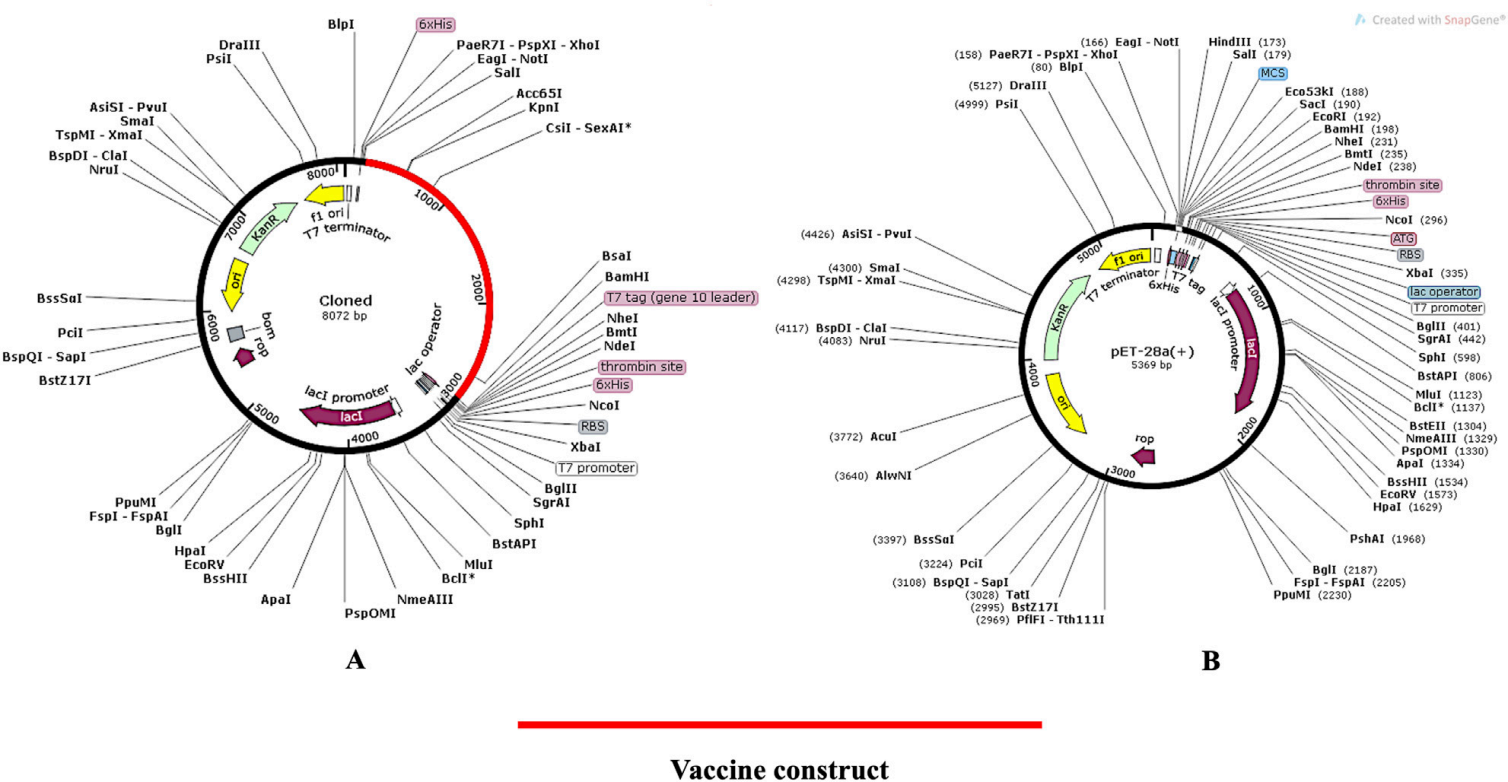
**(A)** In silico cloning of the pET28a (+) vector; **(B)** In silico cloning of the vaccine protein specific optimised codon (red) into the pET28a (+) expression vector. The pET28a (+) backbone is depicted in black, while the vaccine’s gene sequence is shown in red.

The pET (Plasmid for Expression by T7 RNA Polymerase) system is renowned for producing large amounts of protein. Over 220,000 research articles have utilized this system, with more than 40,000 involving pET28a (+). Key advantages include: (i) Strong T7 Promoter: Increases protein production, (ii) Common Restriction Sites: Especially upstream of the T7 promoter, making it ideal for expression cloning, (iii) Lac Operator Regulation: Suppresses uninduced expression, (iv) Self Lac Repressor Encoding: Reduces promoter leakiness, especially beneficial for bacteria with lac repressor deficiencies and (v) Medium Copy Number (20–25 per cell): allows high expression levels without overloading the cell. The proposed vaccine design was cloned *in silico* in the *E. coli* vector backbone following codon optimisation in order to carry out the expression analysis. The codons in the vaccine design were modified to match those in the *E. coli* expression system using the JCat online tool (https://www.jcat.de/). On the Codon Adaptation Index (CAI), a perfect score of 1 was obtained. Similarly, 50.73 percent GC concentration was determined to be optimal for achieving high protein expressions. The pET28 (+) vector was used to clone the vaccine because of its appropriate CAI (1.0) and GC (50.73) content, which indicated a higher likelihood of expression in *E. Coli* cells. The N- and C-terminal portions of the vaccine construct were modified to include XhoI and BamHI restriction sites, as the original build did not have these sites. The cloned map (Figures 10A, B) was visualized and created using the SnapGene software (https://www.snapgene.com/). The resulting restriction clone had a total length of 8,072 base pairs.

### 3.9 Immune simulation for vaccine efficacy

**FIGURE 11:**
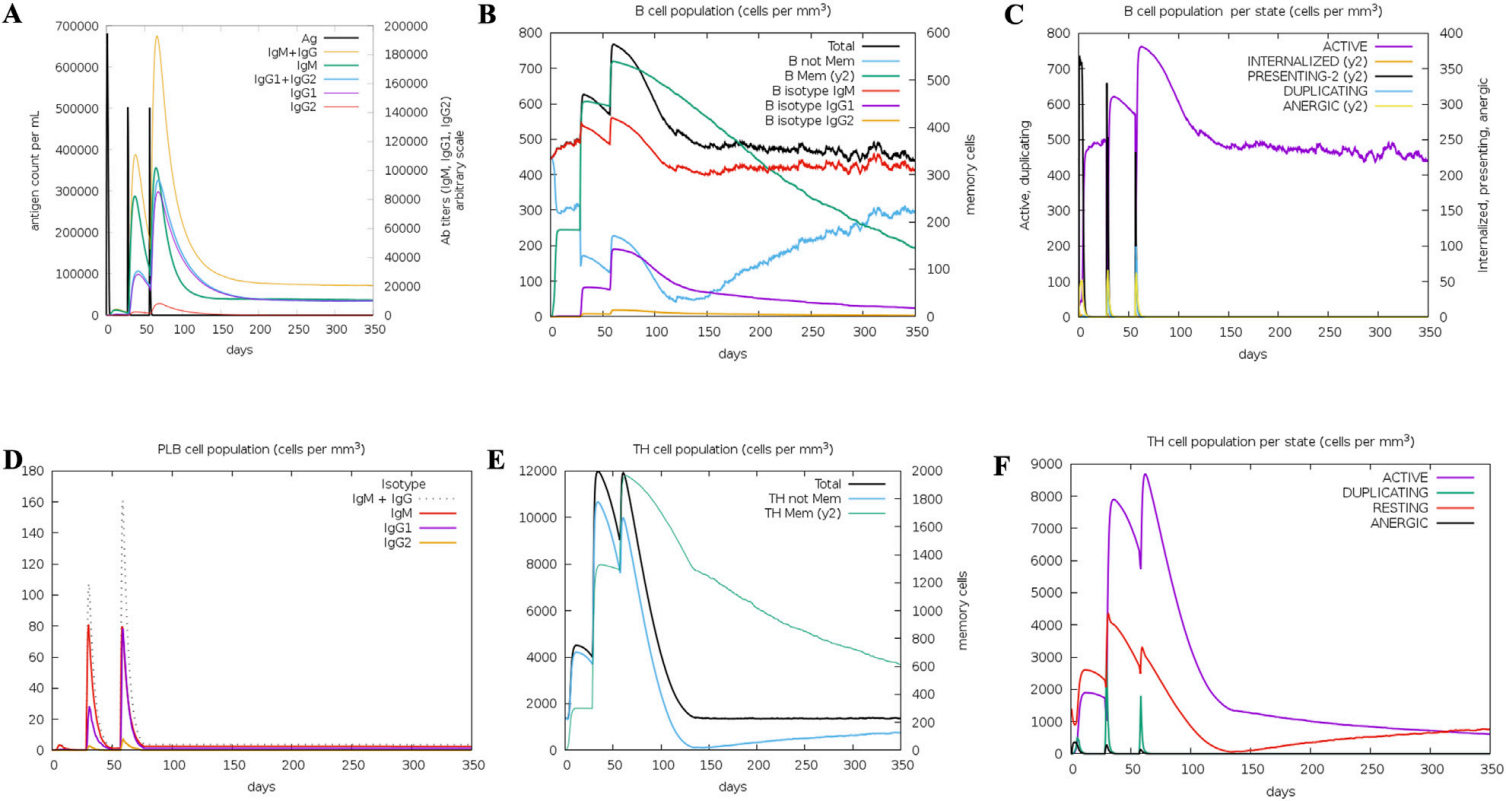
Immunosimulation graphs: **(A)** Represents the Antigen count per ml, **(B)**. Represents B-Cell population (cells per mm^3^), **(C)** Represents B cell population per state (cell per mm^3^), **(D)** Represents PLB cell population (cells per mm^3^), **(E)** Represents TH cell population (cells per mm^3^), **(F)** Represents TH cell population per state (cells per mm^3^).

**FIGURE 12:**
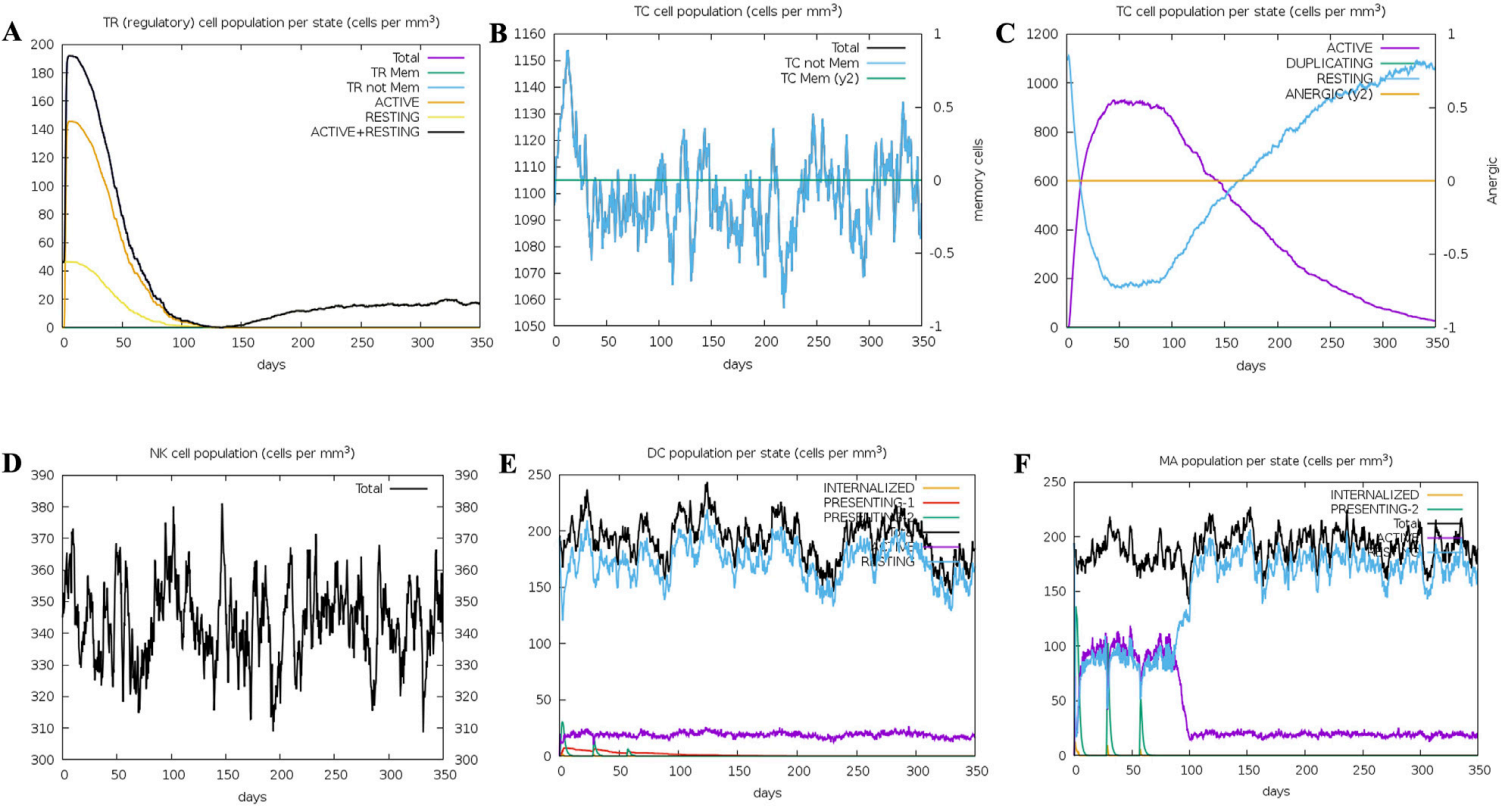
Immunosimulation graphs: **(A)** Represents TR (regulatory) cell population per state (cells per mm^3^) **(B)**. Represents TC Cell population (cells per mm^3^) **(C)**Represents TC cell population per state (cells per mm^3^) **(D)** Represents NK cell population (cells per mm^3^), **(E)** Represents the DC population per state (cells per mm^3^) **(F)** MA population per state (cells per mm^3^).

Several graphs were generated to demonstrate the synthesis of different immune cells in the body, as well as the antigen count per millilitre that caused antibody production. The overall number of B cells, memory cells, and the synthesis of isotypes IgM, IgG1, and IgG2 were all investigated. The B-cell population was evaluated per entity state, which included the number of internalised, presenting, and active antigens, as well as the replicating and anergic states of B-cells. The plasma B-cell (PLB cell) count was estimated for each isotype (IgM, IgG1, and IgG2). The overall count of CD4 T-helper cells, T-regulatory cells, CD8 T-cytotoxic cells, and memory cells was calculated along with the count per entity state showing active, resting, anergic, and replicating state. The concentrations of interleukins and cytokines were tracked using plots that were maintained for 84 days (Figures 11A–F, 12A–F). Compared to the original reaction, the secondary and tertiary responses were substantially bigger. When IgM + IgG, IgM, IgG1 + IgG2, and IgG1 antibodies were present, the antigen concentration dropped (Figure 11A). Figures 11B–F shows an increase in macrophage activity, which is indicative of the vaccine’s ability to provide durable and efficient protection. This resulted in an increase in the quantity of activated B-cells. T helper (Th) (Figures 11A–F) and T cytotoxic cells were found to exhibit similar behaviour (Figure 12). In conclusion, the immune simulation dynamics are consistent with a realistic immunisation process, because they demonstrate a faster secondary response due to the formation of long-lasting protection, as depicted in Figures 11A–F; Supplementary Figures 4A, B.

## 4 Discussion

The giant intestinal fluke *F. buski* is a parasite of major public health relevance as it causes severe illness and mortality, with an estimated 10 million individuals afflicted at the moment. Fasciolopsiasis is underreported and prevalent in rural and semi-urban regions. Human infection, which is more common in children, has been reported in several South and Southeast Asian nations where pigs are the major reservoir of infection, as well as among immigrants from other countries (Mas-coma et al., 2005). Fasciolopsiasis is normally asymptomatic, however eosinophilia is prevalent and can be severe. Flukes can cause temporary blockage and ileus in cases of severe infections. PZQ is the recommended treatment for all intestinal trematode infections. Niclosamide was used to treat fasciolopsis infections, and triclabendazole (TCBZ) may be effective, however experience with this medicine is limited (Maguire, 2015).

The emergence of PZQ and TCBZ resistance in a number of countries, as well as the lack of an effective vaccine to prevent infection, underline the need for the development of new drugs to manage this parasite. To keep the number of vaccinations being tested under control, a combination of logical, empirical, and collaborative approaches are necessary. The emergence of huge omics datasets for *F. buski* has accelerated the identification of key molecules involved in intestinal fluke biology, toxicity, and virulence that could be targeted for vaccine development. Vaccines are crucial for fighting the spread of anthelminthic resistance; yet, only a few drugs have progressed to large animal vaccine studies, particularly in the field (Cwiklinski and Dalton, 2022).

In-silico computer-based techniques increase the efficiency of wet-lab biological experiments. As a result, computational approaches are increasingly being employed to predict antigenic proteins. It has become critical to shift treatment priorities and focus on a long-term treatment strategy that targets the parasitic lifecycle and its various developmental stages, which could potentially give a long-term solution to infection transmission. In order to design a potentially effective chimeric molecule vaccine construct (which is logistically feasible) for the treatment of fasciolopsiasis, the current study aimed at retrieving nine (9) F. buski protein sequences, that included leucine aminopeptidase (LAP), glutathione-S-transferases (GSTs), fatty-acid-binding proteins (FABPs), 14-3-3 epsilon proteins, tegumental calcium-binding EF-hand protein 3&4 and cathepsin B & L peptidases. These sequences were of interest because they are abundantly secreted *in vitro* by the mature adult parasites. By avoiding reactions against other inappropriate epitopes on the antigen, an epitope-based vaccine will provide a more focused immune response against the target antigen (Onile et al., 2020).

Platyhelminth parasites have a streamlined thiol-based redox system where electrons are supplied to oxidised glutathione (GSSG) and thioredoxin (Trx) by a single enzyme called thioredoxin glutathione reductase (TGR), which is a fusion of a glutaredoxin (Grx) domain to canonical thioredoxin reductase (TR) domains. The use of TGR as a schistosomiasis treatment target has been confirmed (Onile et al., 2020). The zymogens that yield cathepsins B and L have an N-terminal propeptide region that functions as a “clamp” to obstruct the peptidase active site, thereby inhibiting the corresponding peptidase. At an acidic pH, propeptide elimination can happen auto- or trans-catalytically (Pritsch et al., 2020; Groves et al., 1998; Stack et al., 2008). It has been proven that the cathepsins B and L of F. hepatica NEJs are found in the gastrodermal cells that line the parasite gut and are released into the low pH environment of the gut lumen, making them suitable vaccine targets. Similarly Leucine aminopeptidase (LAP) of the M17 peptidase family is a useful target antigen for the diagnosis and treatment of several parasitic diseases (schistosomiasis, malaria, fascioliasis) owing to its high immunogenicity.

Helminth parasites produce and secrete a wide range of lipid-binding proteins (LBPs) that help them acquire nutrients from their host, including fatty acids and cholesterol. These LBPs may also disrupt the host’s immune response by sequestering or delivering bioactive lipids. This information necessitates the development of novel therapeutics and disease prevention by these highly pathogenic parasites. The 14-3-3 protein family includes small, acidic proteins that act as scaffolding, chaperones, and adaptors to regulate a variety of intracellular signalling pathways. These proteins are involved in a variety of physiological processes that are currently being investigated for potential pharmacological intervention. The 14-3-3 proteins of *S. mansoni* and *E. multilocularis* have been tested as potential vaccine candidates in mice, and they show promise as defence mechanisms (Siles-Lucas et al., 2008). To fully realise the promise of 14-3-3 proteins as therapeutic targets, more fundamental understanding, as well as improved models and pharmacological possibilities, are required.

Glutathione S-transferases (GSTs; EC 2.5.1.18) are a broad family of multifunctional dimeric enzymes that conjugate reduced glutathione to electrophilic centres in hydrophobic chemical compounds, and their enzymatic activity has been seen in both adult and larval stages of helminths. The helminth GSTs aid in the detoxification of lipid hydroperoxides and carbonyl cytotoxins generated by oxygen-reactive intermediates (ORI). The helminth GSTs can conjugate glutathione to xenobiotic chemicals or bind to antihelminth medicines, making them a potentially vulnerable target in immunotherapy and chemotherapy (Vibanco-Pérez and Landa-Piedra, 1998).

The idea of a Platyhelminthes-specific protein family, which is distinguished by the combination of two N-terminal EF-hands and a C-terminal dynein light chain-like domain, is supported by the f. buski transcriptome data. Calcium signalling proteins represent attractive targets due to the vital nature of properly regulated calcium-mediated signalling and the presence of unusual calcium-binding proteins in helminths. Further family members of schistosomes elicit an IgE response, which is thought to be a sign of protection and has been linked to resistance to reinfection in schistosomiasis. These findings give more evidence that calcium-binding EF-hand proteins play a role in host-parasite interactions and can serve as potential drug targets (Thomas and Timson, 2019).

Lipoprotein A adjuvant was added in frame to vaccine candidate epitopes using suitable linkers to create a potential fasciolopsiasis vaccine candidate. Previously, a potent chimeric molecule vaccine design with immunogenic properties was produced employing the EAAAK, GPGPGP, AAY, and KK linkers (Ayyagari et al., 2022). As a result, we used EAAAK, GPGPG, KK, and AAY linkers to generate our multiepitope antigen for vaccine development. Linker selection is an important aspect of the immunisation process. The stability of the vaccine structure is largely determined by the separation of functional domains, the generation of an extended conformation (flexibility), and protein folding into a desired shape. The most promising potential epitopes were chosen in order to construct the vaccine. KK helped connect B cell epitopes, and the GPGPG linker stimulates HTL responses while preserving the helper and antibody epitopes’ conformation-dependent immunogenicity. By aiding in the creation of sites suitable for binding to the TAP transporter, the AAY linker improves epitope presentation. Lpr works effectively as an adjuvant to increase the antigenecity of the vaccine because it is an agonist of TLR2. Using several bioinformatics techniques, we explored and confirmed the effectiveness of this vaccine design and discovered that it is nontoxic, nonallergic, and thermally stable. It was verified by molecular docking that it binds to a toll-like receptor-2 (TLR-2) (Pecora et al., 2006).

The designed chimeric molecule vaccine candidate was subjected to a variety of analyses to predict physicochemical parameters, secondary and tertiary structure models, disulfide bonds, and interactions with immune cell receptors. According to the findings, the molecular weight of the chimeric molecule vaccine candidate protein was 98.71 kDa with a theoretical isoelectric pI value of 8.74, and it was slightly stable with an instability score of 27.36. The GRAVY score indicates that the chimeric molecule vaccine candidate protein has a hydrophilic property (−0.136) and strong thermotolerance, with an aliphatic index of 76.93. As described in the results section, it was discovered to be highly antigenic, immunogenic, and non-allergenic. This shows that the epitopic vaccination has the capacity to elicit a powerful immune response without triggering allergic responses.

Previous research indicates that in order to create an effective vaccine design, it is crucial to predict the secondary and tertiary structures of vaccine candidates (Meza et al., 2017; Shey et al., 2021; Vibanco-Pérez and Landa-Piedra et al., 1998). The 3D structure prediction provided a complete understanding of the spatial arrangement of important protein components, establishing a strong framework for the study of protein functions, protein constituents, ligand interactions and dynamics. According to Ramachandran Plot analysis, the majority of residues were found in the preferred and approved sectors, with only a small number located in the ignored sector, demonstrating the model’s high degree of overall quality. Validation analysis with Ramachandran plot showed residues mostly found in the favoured and allowed regions with 92.7% score. Hydrogen bonding, RMSF, RG, and RMSD value all contribute to the validity of these findings.

In order to verify that the final structure will bind to TLR2 and trigger an acute immunological response, it was docked against that receptor. Strong binding affinity between the vaccine design and TLR2 complex is necessary for efficient vaccination administration to the body. The vaccine-TLR 2 complex’s stability and underlying interactions were evaluated using molecular dynamic modelling. The construct vaccine was docked with toll receptors 1–4, and it was observed that the interaction between TLR2 and the immunisation complex had the least energy of any. The structure and TLR2 have excellent binding, as evidenced by RMSD, RMSF, Hbond, and RG values. The MMPBSA (Table 6) results and immunostimulant show that the structure of the TLR2 complex is tightly linked and worthy of future exploration. The final CAI value was 1.0, and the GC content was 50.73% or less, which falls within an acceptable range and shows that expression may be higher in the *E. coli* K-12 system.

**TABLE 6:** Electrostatic, Vanderwal and PB energy from MMPBSA.

MMPBSA	
Name	Energy in kj/mol
VDWAALS	−393.5
EEL	−263.9
PB ENERGY	−754.1

Our study’s goal was to use nine proteins to create a chimeric molecule vaccine to protect against fasciolopsiasis infection. The vaccine-TLR2 complex was generated using effective antigenic receptor docking with the vaccine design, which significantly enhanced the accuracy and scope of our research. Immunological simulation of the vaccine design revealed that it can elicit a significant immunological response by producing infections and neutralising antibodies and other immune cells when injected. For a maximal antigen count per millilitre (680,000/mL), the antibody concentration in terms of IgM (10,000/mL), IgG1 (10,000/mL), IgG2 (0/mL), and IgG1+IgG2 (up to 10,000/mL) demonstrated a good response. Studies using computational and immunoinformatics methods indicated that the vaccine design is stable, highly effective, and safe for use as a target against fasciolopsiasis. The experimental evaluation of this chimeric molecule vaccine construct’s immunogenic behaviour is supported by it.

## 5 Conclusion

In this study, we explored different immunoinformatics approaches to predict a vaccine that could be effective against *F. buski*. Despite promising results, these findings require further validation *in vivo* and *in vitro*. Perhaps, given the vast amount of genetic data for intestinal flukes that has grown and evolved as technology has advanced, we might revaluate our approach and propose a more advanced technique in the future for picking up vaccine molecules. A concerted effort is required to assess and re-evaluate these collective data in order to uncover unique elements of *F. buski* biology, which will play a crucial role in future vaccine development. The logistics, statistics, and high cost of large animal trials (sheep, pigs, and, most notably, cattle) preclude our ability to perform even medium-throughput vaccine screens; thus, these studies will necessitate close collaborations with government/agricultural research institutions that have access to appropriate large animal research facilities. Alternatively, we must use an empirical technique and screen as many candidates as feasible, like we did in this study.

## Data Availability

The datasets presented in this study can be found in online repositories. The names of the repository/repositories and accession number(s) can be found in the article/Supplementary Material.

## References

[B1] Abraham M. J. Murtola T. Schulz R. Páll S. Smith J. C. Hess B. (2015). Gromacs: high performance molecular simulations through multi-level parallelism from laptops to supercomputers. SoftwareX 1–2, 19–25. 10.1016/j.softx.2015.06.001

[B3] Altschul S. F. Gish W. Miller W. Myers E. W. Lipman D. J. (1990). Basic local alignment search tool. J. Mol. Biol. 215 (3), 403–410. 10.1016/S0022-2836(05)80360-2 2231712

[B4] Ayyagari V. S. T C. V. K A. P. Srirama K. (2022). Design of a multi-epitope-based vaccine targeting M-protein of SARS-CoV2: an immunoinformatics approach. J. Biomol. Struct. Dyn. 40 (7), 2963–2977. 10.1080/07391102.2020.1850357 33252008 PMC7754933

[B5] Bhatti H. S. Malla N. Mahajan R. C. Sehgal R. (2000). Fasciolopslasis--a re-emerging infection in Azamgarh (Uttar Pradesh). Indian J. Pathol. Microbiol. 43 (1), 73–76. 12583425

[B6] Bibi S. Ullah I. Zhu B. Adnan M. Liaqat R. Kong W. B. (2021). *In silico* analysis of epitope-based vaccine candidate against tuberculosis using reverse vaccinology. Sci. Rep. 11 (1), 1249. 10.1038/s41598-020-80899-6 33441913 PMC7807040

[B7] Biswal D. K. Debnath M. Kharumnuid G. Thongnibah W. Tandon V. (2016a). Northeast India helminth parasite information database (NEIHPID): knowledge base for helminth parasites. PLoS One 11 (6), e0157459. 10.1371/journal.pone.0157459 27285615 PMC4902196

[B8] Biswal D. K. Ghatani S. Shylla J. A. Sahu R. Mullapudi N. Bhattacharya A. (2013). An integrated pipeline for next generation sequencing and annotation of the complete mitochondrial genome of the giant intestinal fluke, Fasciolopsis buski (Lankester, 1857) Looss, 1899. PeerJ 1, e207. 10.7717/peerj.207 24255820 PMC3828612

[B9] Biswal D. K. Konhar R. Debnath M. Tandon V. (2016b). Complete mitochondrial genome annotation of the giant intestinal fluke, *Fasciolopsis buski* (Indian isolate) as revealed by ion torrent and illuminanext-generation sequencing. Mitochondrial DNA B Resour. 1 (1), 693–695. 10.1080/23802359.2016.1222249 34395880 PMC7875049

[B10] Biswal D. K. Roychowdhury T. Pandey P. Tandon V. (2018). *De novo* genome and transcriptome analyses provide insights into the biology of the trematode human parasite Fasciolopsis buski. PLoS One 13 (10), e0205570. 10.1371/journal.pone.0205570 30325945 PMC6191129

[B11] Buchan D. W. A. Jones D. T. (2019). The PSIPRED protein analysis workbench: 20 years on. Nucleic Acids Res. 47 (W1), W402-W407–W407. 10.1093/nar/gkz297 31251384 PMC6602445

[B12] Capron A. Capron M. Dombrowicz D. Riveau G. (2001). Vaccine strategies against schistosomiasis: from concepts to clinical trials. Int. Arch. Allergy Immunol. 124 (1-3), 9–15. 10.1159/000053656 11306914

[B13] Carbone A. Zinovyev A. Képès F. (2003). Codon adaptation index as a measure of dominating codon bias. Bioinformatics 19 (16), 2005–2015. 10.1093/bioinformatics/btg272 14594704

[B14] Chaithirayanon K. Grams R. Vichasri-Grams S. Hofmann A. Korge G. Viyanant V. (2006). Molecular and immunological characterization of encoding gene and 14-3-3 protein 1 in Fasciola gigantica. Parasitology 133 (Pt 6), 763–775. 10.1017/S0031182006001119 16938151

[B15] Chantree P. Phatsara M. Meemon K. Chaichanasak P. Changklungmoa N. Kueakhai P. (2013). Vaccine potential of recombinant cathepsin B against Fasciola gigantica. Exp. Parasitol. 135 (1), 102–109. 10.1016/j.exppara.2013.06.010 23811052

[B16] Cox F. E. (2002). History of human parasitology. Clin. Microbiol. Rev. 15 (4), 595–612. 10.1128/CMR.15.4.595-612.2002 12364371 PMC126866

[B17] Cwiklinski K. Dalton J. P. (2022). Omics tools enabling vaccine discovery against fasciolosis. Trends Parasitol. 38 (12), 1068–1079. 10.1016/j.pt.2022.09.009 36270885

[B20] d'Aubenton Carafa Y. Brody E. Thermes C. (1990). Prediction of rho-independent *Escherichia coli* transcription terminators. A statistical analysis of their RNA stem-loop structures. J. Mol. Biol. 216 (4), 835–858. 10.1016/s0022-2836(99)80005-9 1702475

[B21] Dhanda S. K. Vir P. Raghava G. P. (2013). Designing of interferon-gamma inducing MHC class-II binders. Biol. Direct 8, 30. 10.1186/1745-6150-8-30 24304645 PMC4235049

[B22] Dimitrov I. Bangov I. Flower D. R. Doytchinova I. (2014). AllerTOP v.2 - a server for *in silico* prediction of allergens. J. Mol. Model. 20, 2278. 10.1007/s00894-014-2278-5 24878803

[B23] Doytchinova I. A. Flower D. R. (2007a). Identifying candidate subunit vaccines using an alignment-independent method based on principal amino acid properties. Vaccine 25 (5), 856–866. 10.1016/j.vaccine.2006.09.032 17045707

[B24] Doytchinova I. A. Flower D. R. (2007b). VaxiJen: a server for prediction of protective antigens, tumour antigens and subunit vaccines. BMC Bioinforma. 8, 4. 10.1186/1471-2105-8-4 PMC1780059 17207271

[B25] Ehsan M. Hu R. S. Hou J. L. Elsheikha H. M. Li X. D. Liang P. H. (2021). Fasciola gigantica tegumental calcium-binding EF-hand protein 4 exerts immunomodulatory effects on goat monocytes. Parasit. Vectors 14 (1), 276. 10.1186/s13071-021-04784-5 34022913 PMC8141160

[B26] Greenwood B. (2014). The contribution of vaccination to global health: past, present and future. Philos. Trans. R. Soc. Lond B Biol. Sci. 369 (1645), 20130433. 10.1098/rstb.2013.0433 24821919 PMC4024226

[B27] Grote A. Hiller K. Scheer M. Münch R. Nörtemann B. Hempel D. C. (2005). JCat: a novel tool to adapt codon usage of a target gene to its potential expression host. Nucleic Acids Res. 33 (Web Server issue), W526–W531. 10.1093/nar/gki376 15980527 PMC1160137

[B28] Groves M. R. Coulombe R. Jenkins J. Cygler M. (1998). Structural basis for specificity of papain-like cysteine protease proregions toward their cognate enzymes. Proteins 32, 504–514. 10.1002/(sici)1097-0134(19980901)32:4<504::aid-prot8>3.3.co;2-q 9726419

[B29] Gupta S. Kapoor P. Chaudhary K. Gautam A. Kumar R. Open Source Drug Discovery Consortium (2013). *In silico* approach for predicting toxicity of peptides and proteins. PLoS One 8 (9), e73957. 10.1371/journal.pone.0073957 24058508 PMC3772798

[B30] Heo L. Park H. Seok C. (2013). GalaxyRefine: protein structure refinement driven by side-chain repacking. Nucleic Acids Res. 41 (Web Server issue), W384–W388. 10.1093/nar/gkt458 23737448 PMC3692086

[B31] Hsu P. J. (1964). A survey of fasciolopsiasis of pigs in Kwangtung Province. Acta Veterinaria Zootechnica Sinica 7 (2), 143–150.

[B32] Källberg M. Wang H. Wang S. Peng J. Wang Z. Lu H. (2012). Template-based protein structure modeling using the RaptorX web server. Nat. Protoc. 7 (8), 1511–1522. 10.1038/nprot.2012.085 22814390 PMC4730388

[B33] Kozakov D. Beglov D. Bohnuud T. Mottarella S. E. Xia B. Hall D. R. (2013). How good is automated protein docking? Proteins 81 (12), 2159–2166. 10.1002/prot.24403 23996272 PMC3934018

[B34] Kozakov D. Hall D. R. Xia B. Porter K. A. Padhorny D. Yueh C. (2017). The ClusPro web server for protein-protein docking. Nat. Protoc. 12 (2), 255–278. 10.1038/nprot.2016.169 28079879 PMC5540229

[B35] Laskowski R. A. Jabłońska J. Pravda L. Vařeková R. S. Thornton J. M. (2018). PDBsum: structural summaries of PDB entries. Protein Sci. 27 (1), 129–134. 10.1002/pro.3289 28875543 PMC5734310

[B36] Magnan C. N. Randall A. Baldi P. (2009). SOLpro: accurate sequence-based prediction of protein solubility. Bioinformatics 25 (17), 2200–2207. 10.1093/bioinformatics/btp386 19549632

[B37] Maguire J. H. (2015). Trematodes (schistosomes and liver, intestinal, and lung flukes). Mandell, Douglas, Bennett’s Princ. Pract. Infect. Dis., 3216–3226.e3. 10.1016/b978-1-4557-4801-3.00290-3

[B38] Malecela M. N. Ducker C. (2021). A road map for neglected tropical diseases 2021-2030. Trans. R. Soc. Trop. Med. Hyg. 115 (2), 121–123. 10.1093/trstmh/trab002 33508095 PMC7842088

[B39] Marcilla A. De la Rubia J. E. Sotillo J. Bernal D. Carmona C. Villavicencio Z. (2008). Leucine aminopeptidase is an immunodominant antigen of Fasciola hepatica excretory and secretory products in human infections. Clin. Vaccine Immunol. 15 (1), 95–100. 10.1128/CVI.00338-07 18003812 PMC2223862

[B40] Mas-Coma S. Bargues M. D. Valero M. A. (2005). Fascioliasis and other plant-borne trematode zoonoses. Int. J. Parasitol. 35 (11-12), 1255–1278. 10.1016/j.ijpara.2005.07.010 16150452

[B41] Meza B. Ascencio F. Sierra-Beltrán A. P. Torres J. Angulo C. (2017). A novel design of a multi-antigenic, multistage and multi-epitope vaccine against *Helicobacter pylori*: an *in silico* approach. Infect. Genet. Evol. 49, 309–317. 10.1016/j.meegid.2017.02.007 28185986

[B42] Nakagawa K. (1922). The development of Fasciolopsis buski Lankester. J. Parasitol. 8 (4), 161–166. 10.2307/3271232

[B43] Onile O. S. Fadahunsi A. I. Adekunle A. A. Oyeyemi B. F. Anumudu C. I. (2020). An immunoinformatics approach for the design of a multi-epitope subunit vaccine for urogenital schistosomiasis. PeerJ 8, e8795. 10.7717/peerj.8795 33062404 PMC7534685

[B44] Pecora N. D. Gehring A. J. Canaday D. H. Boom W. H. Harding C. V. (2006). *Mycobacterium tuberculosis* LprA is a lipoprotein agonist of TLR2 that regulates innate immunity and APC function. J. Immunol. 177 (1), 422–429. 10.4049/jimmunol.177.1.422 16785538

[B45] Perera D. J. Ndao M. (2021). Promising technologies in the field of helminth vaccines. Front. Immunol. 12, 711650. 10.3389/fimmu.2021.711650 34489961 PMC8418310

[B46] Pritsch I. C. Tikhonova I. G. Jewhurst H. L. Drysdale O. Cwiklinski K. Molento M. B. (2020). Regulation of the Fasciola hepatica newly excysted juvenile cathepsin L3 (FhCL3) by its propeptide: a proposed ‘clamp-like’ mechanism of binding and inhibition. BMC Mol. Cell Biol. 21, 90. 10.1186/s12860-020-00335-5 33287692 PMC7720491

[B47] Rajendran C. Satbigea A. S. Kumar D. (2019). Review on zoonotic parasitic diseases of northeast India - epidemiology and clinical features. Int. J. Curr. Microbiol. App. Sci. 8 (02), xx. 10.20546/ijcmas.2019.802.xx

[B48] Ridha M. R. Indriyati L. Andiarsa D. Wardhana A. H. (2021). A review of Fasciolopsis buski distribution and control in Indonesia. Vet. World 14 (10), 2757–2763. 10.14202/vetworld.2021.2757-2763 34903937 PMC8654757

[B49] Roberts L. S. Janovy J. Jr (2009). Foundations of parasitology. New York, USA: McGraw Hill, 272–273.

[B50] Roy B. Tandon V. (1993). Morphological and microtopographical strain variations among Fasciolopsis buski originating from different geographical areas. Acta Parasitol. 38, 72–77.

[B51] Saha S. Raghava G. P. (2006a). Prediction of continuous B-cell epitopes in an antigen using recurrent neural network. Proteins 65 (1), 40–48. 10.1002/prot.21078 16894596

[B52] Saha S. Raghava G. P. S. (2006b). AlgPred: prediction of allergenic proteins and mapping of IgE epitopes. Nucleic Acids Res. 34, W202–W209. 10.1093/nar/gkl343 16844994 PMC1538830

[B53] Sanches R. C. O. Tiwari S. Ferreira L. C. G. Oliveira F. M. Lopes M. D. Passos M. J. F. (2021). Immunoinformatics design of multi-epitope peptide-based vaccine against schistosoma mansoni using transmembrane proteins as a target. Front. Immunol. 12, 621706. 10.3389/fimmu.2021.621706 33737928 PMC7961083

[B54] Sen-Hai Y. Mott K. E. (1994). Epidemiology and morbidity of food-borne intestinal trematode infections. Trop. Dis. Bull. 91, r126–r150.

[B55] Sharma N. Singh V. Shyma K. P. (2015). Role of parasitic vaccines in integrated control of parasitic diseases in livestock. Vet. World 8 (5), 590–598. 10.14202/vetworld.2015.590-598 27047140 PMC4774718

[B56] Shey R. A. Ghogomu S. M. Esoh K. K. Nebangwa N. D. Shintouo C. M. Nongley N. F. (2019). In-silico design of a multi-epitope vaccine candidate against onchocerciasis and related filarial diseases. Sci. Rep. 9, 4409. 10.1038/s41598-019-40833-x 30867498 PMC6416346

[B57] Shey R. A. Ghogomu S. M. Shintouo C. M. Nkemngo F. N. Nebangwa D. N. Esoh K. (2021). Computational design and preliminary serological analysis of a novel multi-epitope vaccine candidate against onchocerciasis and related filarial diseases. Pathogens 10 (2), 99. 10.3390/pathogens10020099 33494344 PMC7912539

[B58] Siles-Lucas M. Merli M. Gottstein B. (2008). 14-3-3 proteins in Echinococcus: their role and potential as protective antigens. Exp. Parasitol. 119 (4), 516–523. 10.1016/j.exppara.2008.01.009 18316081

[B59] Singh A. Thakur M. Sharma L. K. Chandra K. (2020). Designing a multi-epitope peptide based vaccine against SARS-CoV-2. Sci. Rep. 10, 16219. 10.1038/s41598-020-73371-y 33004978 PMC7530768

[B60] Smooker P. M. Jayaraj R. Pike R. N. Spithill T. W. (2010). Cathepsin B proteases of flukes: the key to facilitating parasite control? Trends Parasitol. 26 (10), 506–514. 10.1016/j.pt.2010.06.001 20580610

[B61] Stack C. M. Caffrey C. R. Donnelly S. M. Seshaadri A. Lowther J. Tort J. F. (2008). Structural and functional relationships in the virulence-associated cathepsin L proteases of the parasitic liver fluke, Fasciola hepatica. J. Biol. Chem. 283, 9896–9908. 10.1074/jbc.M708521200 18160404 PMC3979170

[B62] Taraschewski H. Mehlhorn H. Bunnag D. Andrews P. Thomas H. (1986). Effects of praziquantel on human intestinal flukes (Fasciolopsis buski and Heterophyes heterophyes). Zentralbl Bakteriol. Mikrobiol. Hyg. A 262 (4), 542–550. 10.1016/s0176-6724(86)80148-1 3799097

[B63] Thomas C. M. Timson D. J. (2019). Characterization of calcium-binding proteins from parasitic worms. Methods Mol. Biol. 1929, 615–641. 10.1007/978-1-4939-9030-6_39 30710301

[B64] Turner J. Howell A. McCann C. Caminade C. Bowers R. G. Williams D. (2016). A model to assess the efficacy of vaccines for control of liver fluke infection. Sci. Rep. 6, 23345. 10.1038/srep23345 27009747 PMC4806326

[B75] Vibanco-Pérez N. Landa-Piedra A. (1998). Glutathione S-transferase in helminth parasites. Revista latinoamericana de microbiologia 40 (1–2), 73–85. 10932736

[B65] Vicente B. López-Abán J. Rojas-Caraballo J. del Olmo E. Fernández-Soto P. Muro A. (2016). Protection against Schistosoma mansoni infection using a Fasciola hepatica-derived fatty acid binding protein from different delivery systems. Parasit. Vectors 9, 216. 10.1186/s13071-016-1500-y 27090442 PMC4836169

[B66] Wang S. Li W. Liu S. Xu J. (2016). RaptorX-Property: a web server for protein structure property prediction. Nucleic Acids Res. 44 (W1), W430–W435. 10.1093/nar/gkw306 27112573 PMC4987890

[B67] Wiederstein M. Sippl M. J. (2007). ProSA-web: interactive web service for the recognition of errors in three-dimensional structures of proteins. Nucleic Acids Res. 35 (Web Server issue), W407–W410. 10.1093/nar/gkm290 17517781 PMC1933241

[B68] Wijffels G. L. Panaccio M. Salvatore L. Wilson L. Walker I. D. Spithill T. W. (1994). The secreted cathepsin L-like proteinases of the trematode, Fasciola hepatica, contain 3-hydroxyproline residues. Biochem. J. 299 (Pt 3), 781–790. 10.1042/bj2990781 8192668 PMC1138089

[B69] Wilkins M. R. Gasteiger E. Bairoch A. Sanchez J. C. Williams K. L. Appel R. D. (1999). Protein identification and analysis tools in the ExPASy server. Methods Mol. Biol. 112, 531–552. 10.1385/1-59259-584-7:531 10027275

[B70] Williams D. L. Bonilla M. Gladyshev V. N. Salinas G. (2013). Thioredoxin glutathione reductase-dependent redox networks in platyhelminth parasites. Antioxid. Redox Signal 19 (7), 735–745. 10.1089/ars.2012.4670 22909029 PMC3739949

[B71] Wu C. H. Yeh L. S. L. Huang H. Arminski L. Castro-Alvear J. Chen Y. (2003). The protein information resource. Nucleic Acids Res. 31, 345–347. 10.1093/nar/gkg040 12520019 PMC165487

