# Supplementary material for: In silico design of an epitope-based vaccine ensemble for fasliolopsiasis
Source: Front Genet. 2025 Jan 22;15:1451853. doi: 10.3389/fgene.2024.1451853 (PMC11794225; doi:10.3389/fgene.2024.1451853)
Supplement: Supplementary file 1 [file DataSheet1.pdf]

In silico Design of an Epitope-Based Vaccine Ensemble for Fasliolopsiasis

Ruchishree Konhar[3], Kanhu Charan Das[1,2], Aiboklang Nongrum[1], Rohan Raj Samal[2], Shailesh Kumar[2], Devendra Kumar Biswal[1,2]*

[1]Department of Zoology, North-Eastern Hill University, Shillong, Meghalaya, India

[2]Bioinformatics Centre, North-Eastern Hill University, Shillong, Meghalaya, India

[3]Informatics and Big Data, CSIR-Institute of Genomics and Integrative Biology, Delhi, India

Availability of data and materials

Sequence Accessions available at NCBI: (KAA0194699.1, KAA0200776.1, KAA0200004.1, KAA0200256.1, KAA0201253.1, KAA0194461.1, KAA0193008.1, KAA0189127.1, and KAA0189126.1) and obtained from NCBI in FASTA format.

Structure of TLR2 (PDB ID: 5D3I) available at NCBI.

Antigenicity: (http://imed.med.ucm.es/Tools/antigenic.pl). ABCPred tools: (https://webs.iiitd.edu.in/raghava/abcpred/ABC_submission.html). IFN-γ Inducer: (http://crdd.osdd.net/raghava/ifnepitope/predict.php). ToxinPred: (http://crdd.osdd.net/raghava/toxinpred/multi_submit.php). Vaxijen: (http://www.ddg-pharmfac.net/vaxijen/VaxiJen/VaxiJen.html). Allertop: (https://www.ddg-pharmfac.net/AllerTOP/). Expaxy Protparam Tools: (https://web.expasy.org/protparam/). Raptor-X server: (http://raptorx.uchicago.edu/StructurePrediction/predict/). Java codon adaption tool: (http://www.jcat.de/). C-IMMSIM (https://kraken.iac.rm.cnr.it/C-IMMSIM/index.php?page=1).

Supplementary Fig.1: (A) Ramachandran plot of the vaccine construct before refinement of vaccine construct, (B) Ramachandran plot of the vaccine construct after refinement.

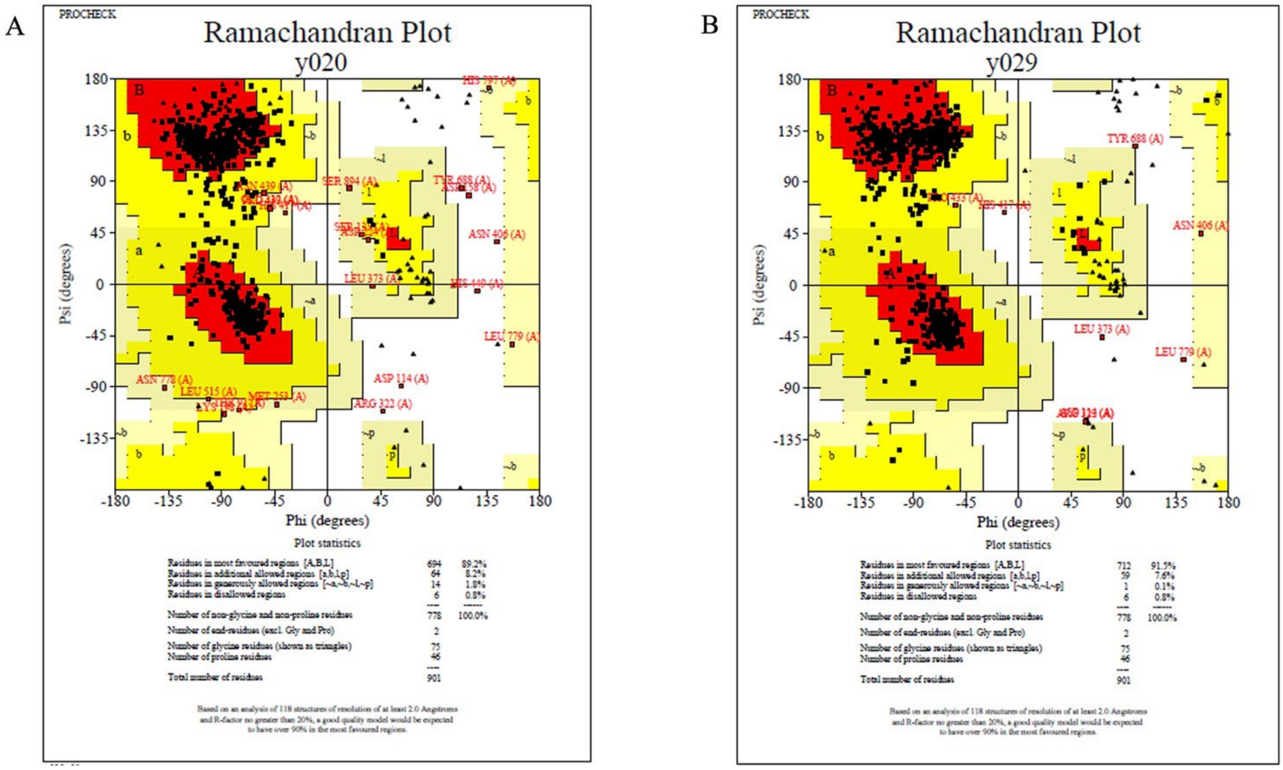

Supplementary Fig.2: (A) Z-score of the vaccine construct before refinement, (B) Z-score of the vaccine construct after refinement, (C) Energy plot of the vaccine construct

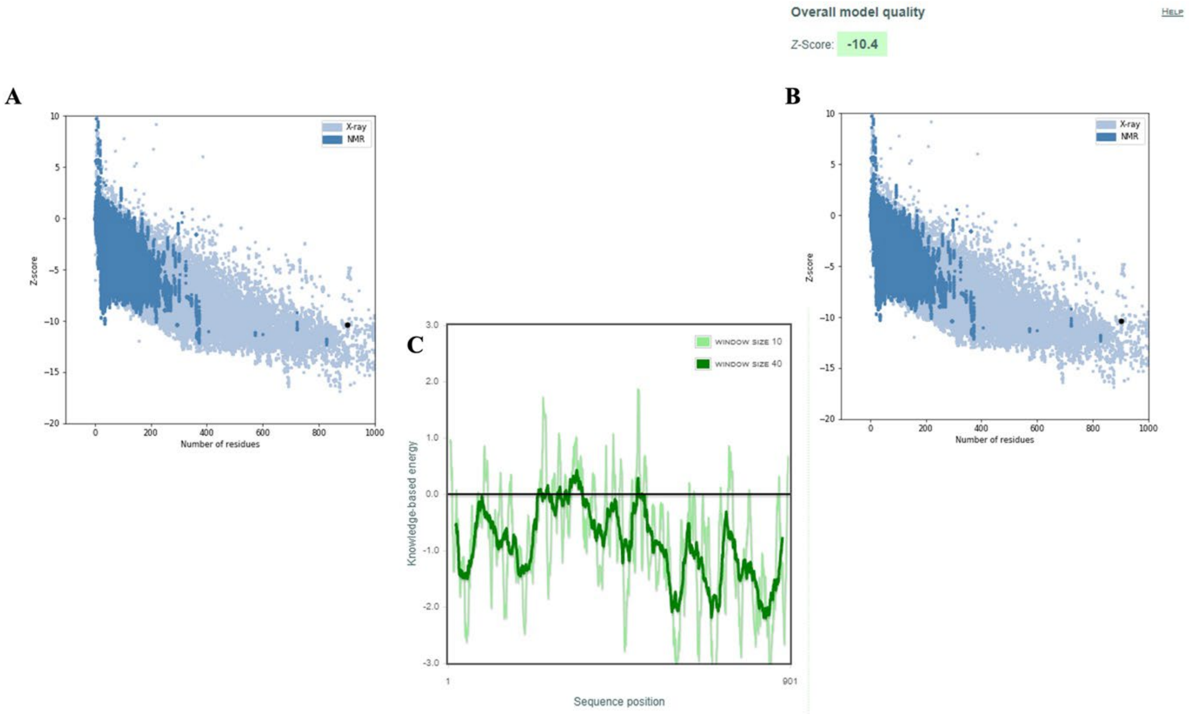

Supplementary Fig. 3: (A) Secondary structure of the vaccine construct, (B) Secondary Structure of the vaccine where red represents the α- helix, pink represents the β-sheet, blue lines represents the coil.

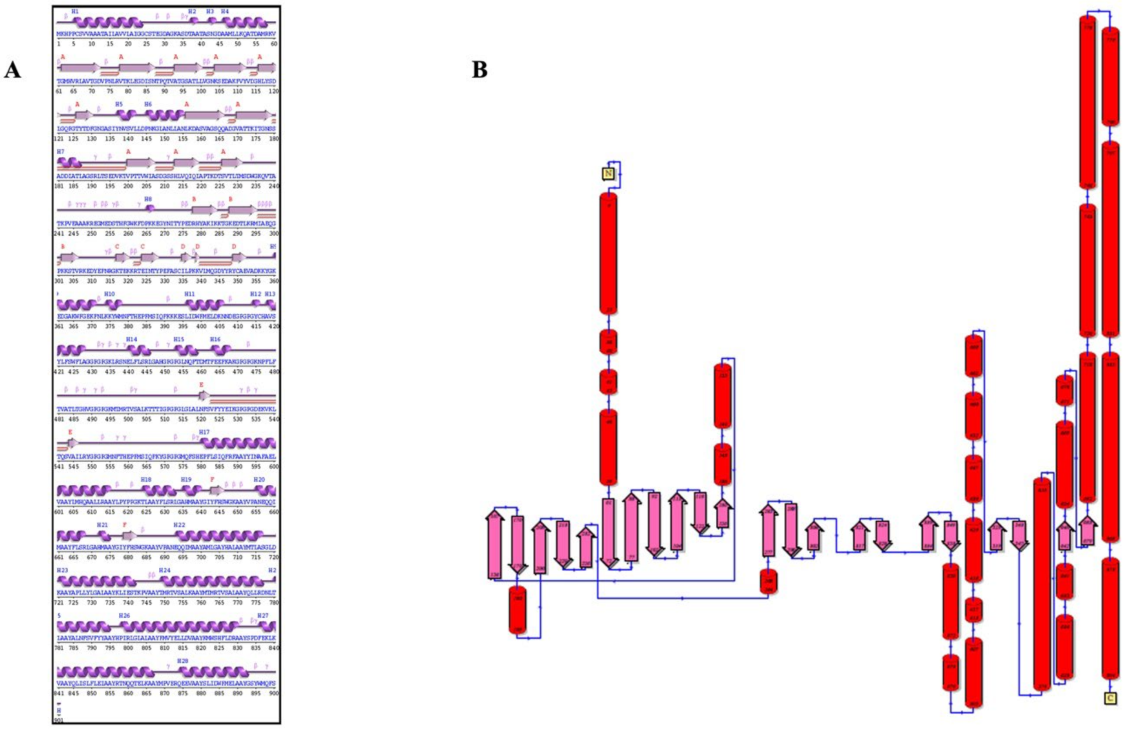

Supplementary Fig. 4: Immunosimulation graphs: (A) EP simulation per state (cells per mm^3), (B) Concentration of cytokines and interleukins.

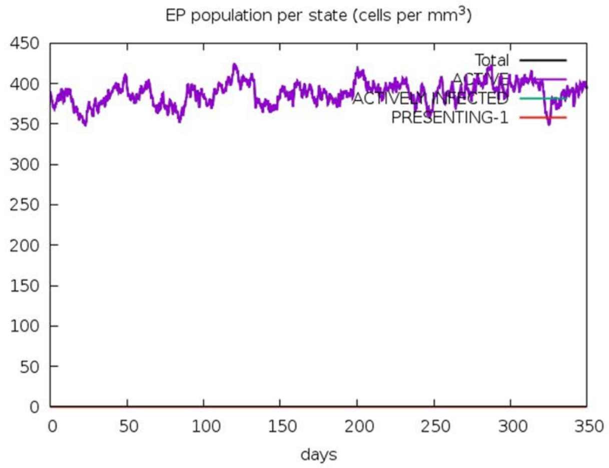

A

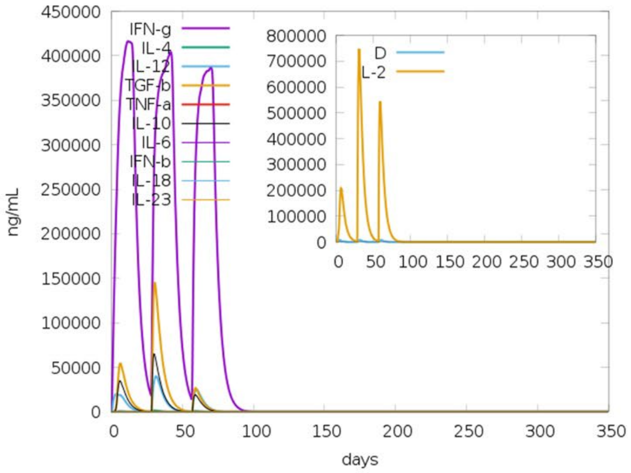

B
